# Unravelling the transcriptomic symphony of muscle ageing: key pathways and hub genes altered by ageing and caloric restriction in rat muscle revealed by RNA sequencing

**DOI:** 10.1186/s12864-024-11051-1

**Published:** 2025-01-13

**Authors:** Gulam Altab, Brian J. Merry, Charles W. Beckett, Priyanka Raina, Inês Lopes, Katarzyna Goljanek-Whysall, João Pedro de Magalhães

**Affiliations:** 1 Integrative Genomics of Ageing Group, Institute of Ageing and Chronic Disease, University of Liverpool, Liverpool, L7 8TX UK; 2 Institute of Integrative Biology, University of Liverpool, Liverpool, L69 3BX UK; 3 College of Medicine, Nursing and Health Sciences, University of Galway, Galway, H91 TK33 Ireland; 4 Institute of Inflammation and Ageing, University of Birmingham, Queen Elizabeth Hospital, Mindelsohn Way, Birmingham, B15 2WB UK

**Keywords:** Sarcopenia, Diet, Functional genomics, Nutrigenomics

## Abstract

Age-related muscle wasting, sarcopenia is an extensive loss of muscle mass and strength with age and a major cause of disability and accidents in the elderly. Mechanisms purported to be involved in muscle ageing and sarcopenia are numerous but poorly understood, necessitating deeper study. Hence, we employed high-throughput RNA sequencing to survey the global changes in protein-coding gene expression occurring in skeletal muscle with age. Caloric restriction (CR) is a known prophylactic intervention against sarcopenia. Therefore, total RNA was isolated from the muscle tissue of both rats fed ad libitum and CR rats. RNA-seq data were subjected to Gene Ontology, pathway, co-expression, and interaction network analyses. This revealed the functional pathways most activated by both ageing and CR, as well as the key “hub” proteins involved in their activation.

RNA-seq revealed 442 protein-coding genes to be upregulated and 377 to be downregulated in aged muscle, compared to young muscle. Upregulated genes were commonly involved in protein folding and immune responses; meanwhile, downregulated genes were often related to developmental biology. CR was found to suppress 69.7% and rescue 57.8% of the genes found to be upregulated and downregulated in aged muscle, respectively. In addition, CR uniquely upregulated 291 and downregulated 304 protein-coding genes. Hub genes implicated in both ageing and CR included *Gc*, *Plg*, *Irf7*, *Ifit3*, *Usp18*, *Rsad2*, *Blm* and *RT1-A2*, whilst those exclusively implicated in CR responses included *Alb*, *Apoa1*, *Ambp*, *F2*, *Apoh*, *Orm1*, *Mx1*, *Oasl2* and *Rtp4*. Hub genes involved in ageing but unaffected by CR included *Fgg*, *Fga*, *Fgb* and *Serpinc1*. In conclusion, this comprehensive RNA sequencing study highlights gene expression patterns, hub genes and signalling pathways most affected by ageing in skeletal muscle. This data may provide the initial evidence for several targets for potential future therapeutic interventions against sarcopenia.

**Supplementary Information:**

The online version contains supplementary material available at 10.1186/s12864-024-11051-1.

## Introduction

Sarcopenia, the age-related reduction in muscle mass accompanied by a decline in strength and function, elevates the likelihood of various adverse clinical outcomes such as disability, falls, and mortality [1–3] and is said to affect around 10% of those over 65 and half of those over 80 [4]. Since its inclusion in the World Health Organisation’s revision of the International Statistical Classification of Diseases and Related Health Problems in 2016 (ICD-11), there has been a surge of research interest in the disease [5, 6]. However, the causes of muscle ageing are complex and enigmatic. Implicated mechanisms include satellite cell exhaustion [7, 8], reduced proteostasis capacity [9], increased ROS production [10], increased inflammation [11], and mitochondrial dysfunction [12]. Understanding both the mechanisms involved in the ageing process, as well as how it may be ameliorated, is crucial to improving the quality of life of elderly patients affected by sarcopenia.

Given its high degree of network complexity, -omics approaches are likely best placed to explain the underlying causal structure of muscle ageing [13]. Previous studies have assessed the transcriptomic signature of muscle ageing in mice [14–16] and human [17]. Caloric restriction has repeatedly been demonstrated to be an effective prophylactic against age-related muscle mass loss in laboratory rodents [18–20] and non-human primates [21–23]. No studies to our knowledge, however, have assessed the transcriptomic correlates of CR-mediated delay of muscle ageing in rats. Using rat tissue, we aimed to replicate previous findings regarding the key changes in protein-coding gene expression observed in muscle ageing. In addition, we endeavoured to assess to what extent these changes could be reversed using CR. Furthermore, we employed Gene Ontology (GO) and Reactome pathway analyses to assess in which functional pathways identified genes were implicated. Finally, we used gene co-expression and protein–protein interaction (PPI) network analyses to identify the most important “hub” genes mediating the effects of both ageing and CR in rat muscle. A previous study has shown that the rat is an appropriate model for muscle ageing, as more age-related genes were identified in rat skeletal muscles compared to mice, and rat muscles undergo a more robust age-related decline in mass. This includes downregulated pathways, involving metabolism and mitochondrial respiration, mirroring human muscle ageing. The pattern of decline in the rat may be more representative of humans [24]. Therefore, based on these findings, the rat was chosen as the model for the current study investigating muscle ageing and sarcopenia.

## Materials and methods

### Animals

Gastrocnemius muscle tissue samples were collected from rats used in a previous study conducted by [25], under Home Office Project License PPL 40/2533. All animal experiments were conducted in compliance with the United Kingdom Animals (Scientific Procedures) Act of 1986. In brief, 21–28 day old, male, Brown Norway (Substrain BN/SsNOlaHSD) rats were acquired from Harlan Laboratories, UK. Animals were kept under barrier conditions on a 12-h light:12-h dark cycle (08:00–20:00). Prior to 2 months of age, rats were group housed (*n* = 4) and fed ad libitum (diet procured from Special Diet Systems Division Dietex International in Witham, UK). Following maturation, all animals were moved to solitary housing and randomly assigned to either ad libitum (AL) or caloric restriction (CR) diet groups. The CR rats were fed the same diet as the ad libitum rats, but their daily intake was restricted to 55% of the age-matched ad libitum intake. The CR rats were given access to food for a limited time each day between 10:30 and 11:00 h, while the ad libitum rats had unlimited access to the food for 24 h per day [25].

Animals were either terminated at 6 or 28 months of age (Table 1). No animals displayed any signs of clinical pathology prior to sacrifice. Following termination, gastrocnemius muscle tissue was removed, flash-frozen in liquid N_2_ and stored at − 80 °C, prior to further analysis.

**Table 1:** Group Identity, Diet Regimen and Age at Termination. Tissue samples were collected from male Brown Norway rats from one of 3 groups (*n* = 6). Following animal arrival at 21–28 days of age, all animals were fed ad libitum (AL). After reaching full maturation/2-months old, animals were randomly allocated to either an AL (groups 1–2) or caloric restriction (CR) diet (group 3). Half the AL-fed animals were terminated at 6-months old and half at 28-months old. CR rats were terminated at 28-months old. Tissue samples were collected immediately post-sacrifice, snap frozen and stored at −80 °C until the day of analysis

Group	Diet	Age at Termination (months)	Number of Animals
1	Ad Libitum	6	6
2	Ad Libitum	28	6
3	Caloric Restriction	28	6

### RNA extraction and RNA‐seq analysis

Following removal from storage, gastrocnemius muscle tissues were immediately transferred onto ice to prevent thawing and degradation. Subsequently, each tissue was ground into a powder using a mortar and pestle. Liquid nitrogen was added periodically to ensure tissues remained in their frozen state. Total RNA was isolated using RNeasy Plus Universal Mini Kit (Qiagen, UK) according to manufacturer instructions. Extracted RNA quantity and quality were assessed on nanoDrop 2000 spectrophotometer and using the Agilent 2100 Bioanalyser, revealing all extracted RNA sampled to be of high quality (RINs > 8). RNA integrity was further examined using agarose gel electrophoresis. Ribosomal RNA (rRNA) was removed from the total RNA using Illumina Ribo-Zero Plus rRNA depletion kit following the manufacturer's instructions. Strand-specific RNA-seq library preparation was done using NEBNext® Ultra™ Directional RNA Library Prep Kit for Illumina, according to manufacturer protocol. High‐throughput RNA‐seq was performed at the Centre for Genomic Research at the University of Liverpool on the Illumina Hiseq 4000 platform (Paired-end, 2 × 150 bp sequencing, generating data from > 280 M clusters per lane). The raw Fastq files were trimmed for the presence of Illumina adapter sequences using Cutadapt version 1.2.1. The reads were further trimmed using Sickle version 1.200 with a minimum window quality score of 20. Lastly, short reads < 20 bp in length were removed.

The filtered reads were mapped to the rat reference genome (https://ftp.ensembl.org/pub/release-100/fasta/rattus_norvegicus/dna/) using STAR 2.3.0e. MultiQC showed the alignment score was above 80% for all the samples. To count the number of reads that mapped to each gene, featureCount was used to obtain the read count. Ensembl Rattus_norvegicus.Rnor_6.0.96.gtf were chosen as annotation references for the analyses.

### Differential expression analysis

Differentially expressed genes (DEGs) were identified by using the Bioconductor package “DESeq2” (version, 3.7) in R (version 4.1.0). Raw gene counts, detected by featureCount, were used as the input. DESeq2 is a well-known method, which offers statistical procedures for identifying differential expression by using negative binomial linear models for individual genes and uses the Wald test for significance analysis. The *P*-values from the Wald test were then adjusted using the Benjamini and Hochberg’s method for controlling the false discovery rate (p-adj). Differentially expressed genes were defined as those with a padj-value <  = 0.05 and fold change (FC) >  = 1.5. Biomart (https://www.ensembl.org/biomart) was used to annotate the biotype of each gene.

### Gene Ontology and pathway analysis

For the GO analysis, we analysed distinct sets of differentially expressed genes separately. Initially, we compared gene expression profiles between young and old samples, delving into the functional implications of both upregulated and downregulated genes in the ageing process. Subsequently, we explored the differentially expressed genes between young and old samples under caloric restriction, investigating their potential roles in ageing under CR conditions. Using Venn diagrams, we further classified these genes into specific groups: those exclusively upregulated or downregulated in either old or old CR muscle, genes suppressed by CR in old muscle, or genes uniquely differentially expressed in old CR muscle. For each subgroup, we conducted Gene Ontology analysis to elucidate their functional significance in the context of ageing and CR.

Both GO and Reactome pathway enrichment analyses were performed using ClusterProfiler (version 3.14.3), which enabled visualization of functional profiles [26]. For this study, we performed GO biological process (GO-BP) analysis in the general level (GO level 3–8). Revigo (http://revigo.irb.hr) was used to remove redundant GO terms, clustering them based on semantic similarity. Input gene sets compared to the background (total expressed genes in muscle samples) by the hypergeometric test were used to obtain the significant GO terms and Reactome pathways (*p* < 0.05) and the *p*-values corrected for false discovery rate (FDR)( padj) <  = 0.05 in multiple testing, q-values are also projected for FDR control. The top 20 GO and Reactome pathways were then visualised using ggplot2 (version 3.3.5) in R.

### Co-expression network construction

Using the weighted gene co-expression network analysis (WGCNA) package in R, an unsigned gene co-expression network was produced [27]. Briefly, an adjacency matrix (Adj) was created from the input gene list by calculating Pearson correlations (Adj =|0.5 x (1 + Corr)| ß) between all pairs of genes among all the provided samples (*n* = 6/ group). Adj was then converted to Topological Overlap Matrix (TOM) using the TOMsimilarity function in R. TOM represent the inter-connectedness between a pair of gene, which was then used to create a network tree through hierarchical clustering. Gene clusters with high topological overlap were designated as a module. Using the dynamicTreeCut R package, modules were detected from the dendrogram and were labelled with a standard colour scheme [28]. The expression profile of genes in each module were represented by module eigengene (ME). The correlation between module eigengene and the gene expression profile was calculated to obtain module membership (MM), and the correlation between the gene profile and the trait (age, e.g., 6 months and 28 months old) was computed to attain Gene Significance (GS). Then, associations between module membership (MM) vs. gene significance (GS) were calculated. GS and MM values were then used to identify hub genes in modules that were strongly correlated with traits of interest, using stringent thresholds (GS values > 0.80 and MM values > 0.80). Finally, interesting modules were visualized by Cytoscape software and further analysed by protein–protein interaction (PPI) network analysis. Weighted Gene Correlation Network Analysis (WGCNA) was conducted using normalised counts of differentially expressed genes from old or old CR rat muscle to identify hub genes associated with muscle ageing or CR.

### Protein–Protein Interaction (PPI) network analysis

To construct PPI networks “The search tool for retrieval of interacting genes” (STRING) (https://string-db.org) was used [29]. The STRING database (version 11.0) allows the identification of known and predicted interaction between proteins, given genes of interest. A stringent threshold interaction score of > 0.7 was used to construct the PPI networks. Networks were downloaded and visualised in Cytoscape software (version 3.9.1). Using cytoHubba (version 0.1), a plugin of Cytoscape, hub proteins/genes were identified [30] using 3 different methods, the maximal clique centrality (MCC) method, maximum neighbourhood component (MNC) method and Degree (Deg) method. This facilitated the identification of the top ten hub genes associated with ageing and CR [30]. Then, Venn diagrams were generated using Venny 2.1 (https://bioinfogp.cnb.csic.es/tools/venny/) to visualise which hub genes overlapped between the 3 methods [31]. Lastly, the overlap between age-associated and CR-associated hub genes was assessed.

### RT-qPCR validation of RNA-seq data

To validate RNA-seq data results, reverse transcriptase quantitative PCR (RT-qPCR) analysis was used. Validation was restricted to a minimum of 3 samples per condition (*n* =  > 3) for whom muscle RNA was still available. Six RNAs that were differentially expressed in old muscle were chosen for validation. These included *Foxo1* (p-adj = 0.0004), *Ogdh* (p-adj = 0.0001), *Klf4* (*p* = 0.005), *Pgc1a* (p-adj = 0.001), *Cry1* (p-adj = 0.00001), and *Nr4a3* (p-adj = 0.000000002) mRNAs.

RNeasy Plus Universal Mini Kit (Qiagen, UK) was used according to the manufacturer's protocol, to isolate the total RNA. Extracted RNA quantity and quality were assessed on the nanoDrop 2000 spectrophotometer and using the Agilent 2100 Bioanalyser. The miRCURY LNA RT Ki (Qiagen, UK) was used to synthesise cDNA and miRCURY LNA RT Kit (Qiagen, UK) was used to perform qPCR, according to manufacturer's protocol in Roche ® LightCycler® 480. F-box protein 45 (Fbxo45) was identified as a relevant housekeeping gene using geNORM [32] thus, used as an internal control for RNA template normalization, F: AAGTCAACGGCAGCTTCCCACA, R: CCAGGAACTCATACCCACGCTC (Origene, USA). Primers were obtained from previous literature or previously validated in our lab. *Foxo1*, F: AATTTGCTAAGAGCCGAGGA, R: AAGTCATCATTGCTGTGGGA [33]. *Nr4a3*, F: AGACAAGAGACGTCGAAATCGAT, R: CTTCACCATCCCGACACTGA [34]. *Ogdh*, F: GAGCTGAACAGGAGACAGGTAT, R: CGTCCTAATTGCTGCTGGTT. *Ppargc1a*, F: AGGTCCCCAGGCAGTAGAT, R: CGTGCTCATTGGCTTCATA [35]. *klf4*, F: CAGCTGGCAAGCGCTACA, R: CCTTTCTCCTGATTATCCATTC [36]. *Cry1*, TTCGCCGGCTCTTCCAA, R: ATTGGCATCAAGGTCCTCAAGA [37]. The delta-delta Ct method (2–∆∆Ct method) was used to calculate the relative expression levels. The result mean ± SEM were plotted using Prism (version, 5.01) for Windows. One-way ANOVA (analysis of variance) and Tukey's Multiple Comparison post-hoc test was conducted to compare the difference between the groups. The null hypothesis of no model effects was accepted at *P* < 0.05.

## Results

### Age-related gene expression changes in the gastrocnemius muscle of AL-fed and CR rats

DESeq2 revealed 819 protein-coding genes to be differentially expressed in the gastrocnemius muscle of aged (28-month-old) rats fed AL when compared to young (6-month-old) rats fed AL. Of these, 442 genes were upregulated, whilst 377 were downregulated, in aged muscle, compared to young muscle. Genes with the greatest degree of up-regulation included *Dclk1*, *Serpina12*, *RT1-A2, Spg21, Igtp, Lgals1, B2m, Ctxn3, Bid* and *Nlrc5* (Table 2A). Genes with the greatest degree of down-regulation included *Kcnc1, Nr4a2, Arntl, Nr4a3, Col1a1, Maf, Mpp3, Auts2, Ddit4* and *Padi2* (Table 2B).

**Table 2:** Genes with the largest magnitude of differential expression in the muscle of aged rats fed ad libitum (AL), when compared to the muscle of young rats fed AL. The tables list the gene most **A.** upregulated and **B.** downregulated by ageing. Differential expression was defined as a padj-value <  = 0.05 and fold change >  = 1.5. Adjusted by padj

Symbol	log_2_(FoldChange)	padj	Gene Name
**A**			
Dclk1	2.80	6.08E-20	Doublecortin like kinase 1
Serpina12	4.02	4.83E-14	Serpin family A member 12 (Vaspin)
RT1-A2	1.56	1.12E-12	Rat MHC class I
Spg21	0.93	1.54E-12	Spastic paraplegia 21 (Mast syndrome)
Igtp	1.01	2.14E-11	Interferon gamma induced GTPase
Lgals1	0.78	5.60E-10	Galectin-1
B2m	1.27	7.68E-10	Beta-2-microglobulin
Ctxn3	2.86	2.11E-09	Cortexin 3
Bid	1.16	3.09E-09	BH3 interacting domain death agonist
Nlrc5	2.10	3.35E-09	NLR family CARD domain-containing 5
**B**			
Kcnc1	−2.81	4.02E-17	Potassium voltage-gated channel subfamily C member 1
Nr4a2	−3.00	1.24E-13	Nuclear receptor subfamily 4 group A member 2
Arntl	−1.32	8.92E-11	Aryl hydrocarbon receptor nuclear translocator-like (BMAL1)
Nr4a3	−2.86	1.59E-09	Nuclear receptor subfamily 4 group A member 3
Col1a1	−1.82	2.11E-09	Collagen type I alpha 1 chain
Maf	−1.23	3.05E-09	Avian musculoaponeurotic fibrosarcoma oncogene homolog
Mpp3	−1.91	4.50E-09	Membrane palmitoylated protein 3
Auts2	−1.77	1.35E-08	Autism susceptibility candidate 2
Ddit4	−1.46	2.48E-08	DNA damage-inducible transcript 4
Padi2	−1.74	3.79E-08	Peptidyl arginine deiminase type 2

DESeq2 revealed 888 protein-coding genes to be differentially expressed in the gastrocnemius muscle of aged CR rats when compared to young rats fed AL. Of these, 425 were upregulated, whilst 463 were downregulated, in CR aged muscle, compared to young muscle. Genes with the greatest degree of up-regulation included *Dbp, Txndc16, Cst7, Per3, Hook1, Cpt1a, Rusc2, Slc7a2, Tef* and *Adamts20* (Table 3A). Genes with the greatest degree of down-regulation included *Arntl, Ubc, Slc45a3, Slc41a3, Npas2, Tead4, Asb2, Nfil3, Dyrk2* and *Smtnl2* (Table 3B).

**Table 3:** Genes with the largest magnitude of differential expression in the muscle of aged calorically restricted rats when compared to the muscle of young rats fed ad libitum. The tables list the gene most **A.** upregulated and **B.** downregulated by exposure to caloric restriction. Differential expression was defined as a padj-value <  = 0.05 and fold change >  = 1.5.Adjusted by padj

Symbol	log_2_(FoldChange)	padj	Gene Name
**A**			
Dbp	2.73	4.09E-47	D-box binding PAR bZIP transcription factor
Txndc16	1.19	1.81E-38	Thioredoxin domain containing 16
Cst7	3.11	8.64E-19	Cystatin F (also known as Cystatin-7)
Per3	1.41	1.24E-15	Period circadian regulator 3
Hook1	1.80	1.24E-15	Hook microtubule tethering protein 1
Cpt1a	1.18	2.10E-15	Carnitine palmitoyltransferase 1A
Rusc2	1.49	1.09E-14	RUN and SH3 domain containing 2
Slc7a2	1.36	5.31E-14	Solute carrier family 7 member 2
Tef	0.97	2.13E-13	Thyrotrophic embryonic factor
Adamts20	1.50	1.26E-12	ADAM metallopeptidase with thrombospondin type 1 motif 20
**B**			
Arntl	−3.10	1.05E-75	Aryl hydrocarbon receptor nuclear translocator-like 1 (BMAL1)
Ubc	−1.60	1.77E-27	Ubiquitin C
Slc45a3	−1.42	5.28E-24	Solute carrier family 45 member 3
Slc41a3	−1.90	2.69E-20	Solute carrier family 41 member 3
Npas2	−2.38	2.91E-20	Neuronal PAS domain protein 2
Tead4	−1.32	1.51E-18	TEA domain transcription factor 4
Asb2	−1.24	2.10E-15	Ankyrin repeat and SOCS box containing 2
Nfil3	−1.12	9.66E-15	Nuclear factor, interleukin 3 regulated
Dyrk2	−1.74	1.22E-12	Dual-specificity tyrosine-(Y)-phosphorylation-regulated kinase 2
Smtnl2	−1.19	3.00E-12	Smoothelin-like 2

### Effect of CR on age-related changes in gene expression in the rat gastrocnemius muscle

Comparison of aged rats fed AL with those that were CR, revealed CR to ameliorate age-related changes in the expression of 516 protein-coding genes. Of the gene found to be upregulated in aged muscle 308 (69.7%) were suppressed by CR, whilst 134 (30.3%) were unaffected caloric restriction. Of the gene found to be downregulated in aged muscle 218 (57.8%) were rescued by CR, whilst 159 (42.2%) were unaffected caloric restriction.

Meanwhile, 208 (32%) of the gene found to be downregulated in aged muscle were suppressed by CR. In addition, 291 and 304 genes were respectively upregulated and downregulated uniquely by CR.

### Effect of CR on age-related changes in gene expression in the rat gastrocnemius muscle

**Fig. 1:**
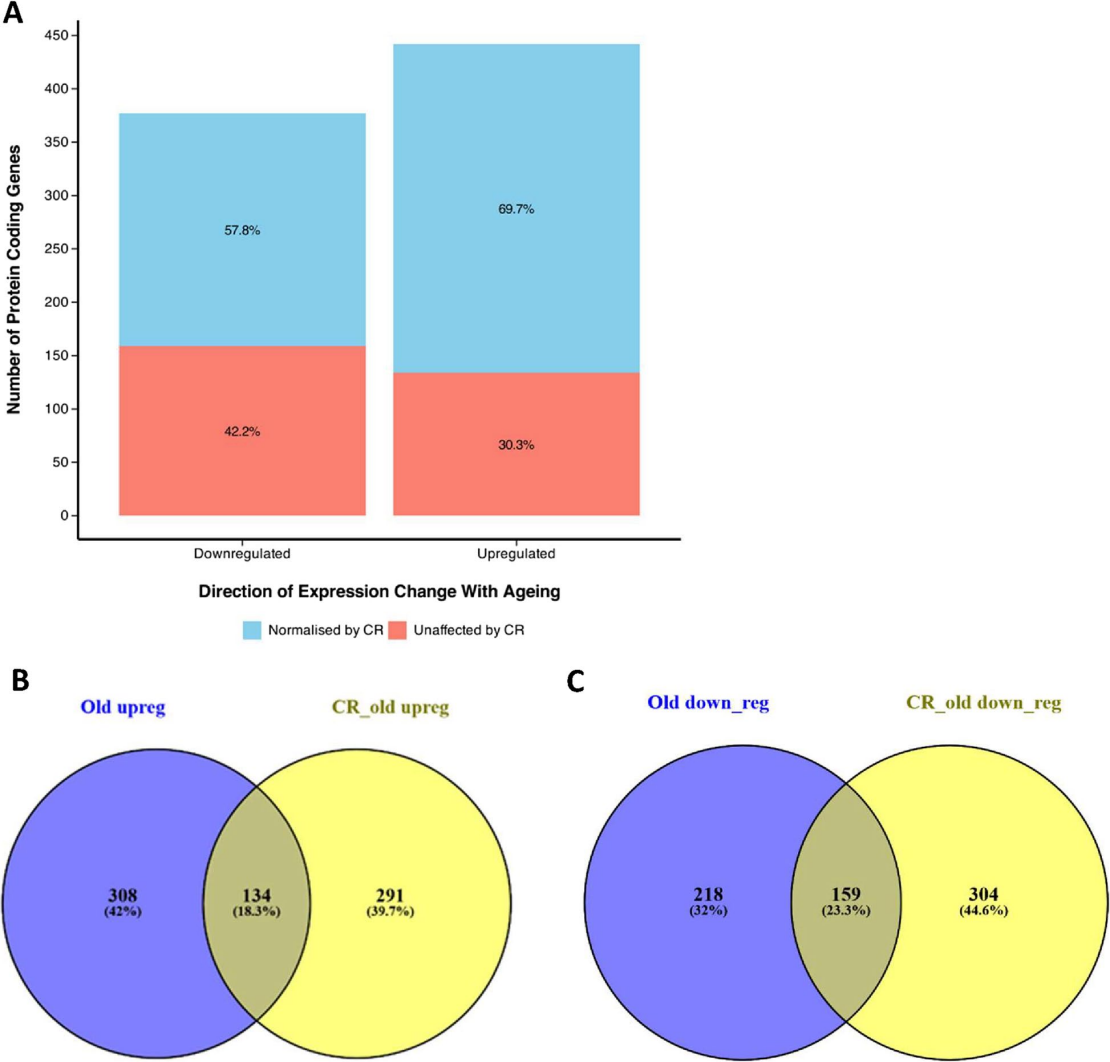
Impact of CR on gene expression. **A** A bar chart showing the number of genes upregulated and downregulated in the muscle of aged rats fed ad libitum (AL), when compared to the muscle of young rats fed AL, and what percentage of these are normalised by caloric restriction (CR). Venn diagrams describe the number of genes **B** Upregulated and **C** Downregulated uniquely in old rats fed AL (purple), uniquely in old CR rats (yellow) and in both groups (centre), when compared to young rats fed AL. Differential expression was defined as FC >  = 1.5-fold, and padj <  = 0.05. Venn diagrams were created using “ https://bioinfogp.cnb.csic.es/tools/venny/”

Comparison between the muscle of aged rats fed AL and aged CR rats, revealed CR to ameliorate age-related changes in the expression of 526 protein-coding genes (Fig. 1). Of the genes found to be upregulated in aged compared to young rats fed AL, 308 (69.7%) were suppressed in aged CR rats, whilst 134 (30.3%) were unaffected. Of the genes found to be downregulated in aged compared to young rats fed AL, 218 (57.8%) were rescued in aged CR rats, whilst 159 (42.2%) were unaffected. In addition, 291 and 304 protein-coding genes were respectively upregulated and downregulated uniquely by CR.

### Functional and pathway analysis of protein-coding genes with differential expression in aged rats fed AL

**Fig. 2:**
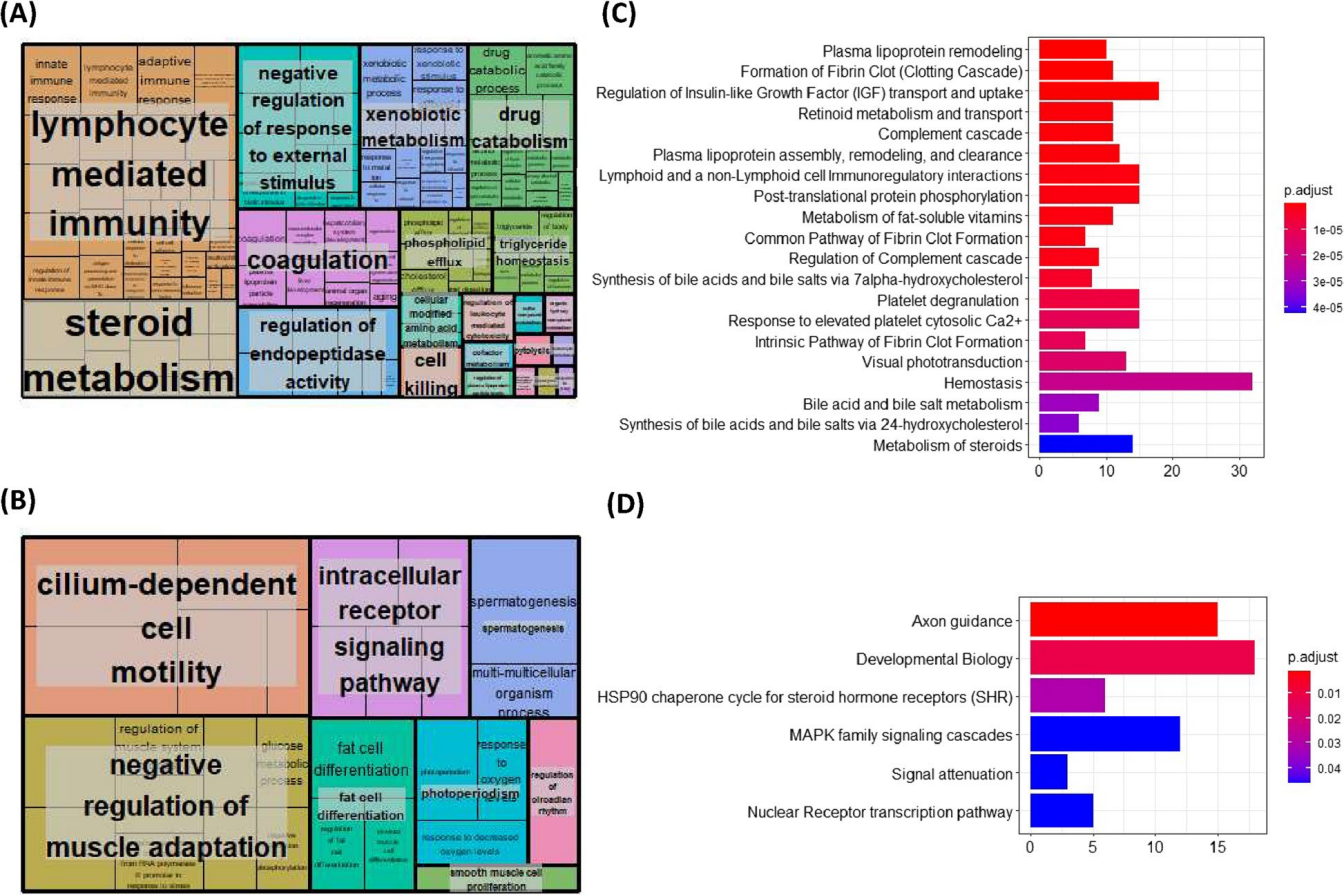
Gene Ontology (GO) and Reactome pathway analysis of genes differentially expressed downregulated in aged compared to young rat muscle. ClusterProfiler (version 3.14.3) package for R, was used to perform GO enrichment analysis. All the genes that were detect in skeletal muscle by RNA-seq analysis was used as background. A padj < 0.05 was used as the cut off *p*-value. Revigo (http://revigo.irb.hr) then summarised GO terms for **A** upregulated genes and **B** downregulated genes. Subsequently, the ReactomePA (version 3.15) package was used for pathway analysis in R software (version 4.1), revealing Reactome pathways significantly (p-adj value =  < 0.05) associated with **C** upregulated and **D** downregulated genes

ClusterProfiler Gene Ontology (GO)-analysis revealed 456 GO terms to be significantly associated with the genes upregulated in aged compared to young rats fed AL. Top upregulated terms based on number of genes included innate immune response, adaptive immune response and cell killing. Revigo summarised these upregulated genes to be primarily involved in lymphocyte-mediated immunity, steroid metabolism, cell killing, negative regulation of responses to external stimuli and coagulation (Fig. 2A). Subsequently, upregulated genes were found to be significantly enriched for 48 Reactome pathways, including plasma lipoprotein remodelling, regulation of insulin-like growth factor (IGF) transport and uptake by binding proteins, and post-translational protein phosphorylation amongst others (Fig. 2C).

ClusterProfiler revealed 40 GO terms to be significantly associated with the genes downregulated in aged compared to young rats fed AL. Top downregulated terms based on gene size included spermatogenesis, negative regulation of carbohydrate metabolism and negative regulation of skeletal muscle cell adaptation, fat cell differentiation, nucleotide catabolic process and skeletal muscle cell differentiation. Revigo summarised downregulated genes to primarily be involved in cilium-dependant motility, negative regulation of muscle adaptation, intracellular signalling and fat cell differentiation (Fig. 2B). Subsequently, downregulated genes in old CR rats were found to be significantly enriched for 6 Reactome pathways. These pathways included axon guidance, developmental biology (including myogenesis), HSP90 chaperone cycle for steroid hormone receptors (SHR), MAPK family signalling cascades and nuclear receptor transcription pathways (Fig. 2D).

### Functional and pathway analysis of protein-coding genes normalised by CR

**Fig. 3:**
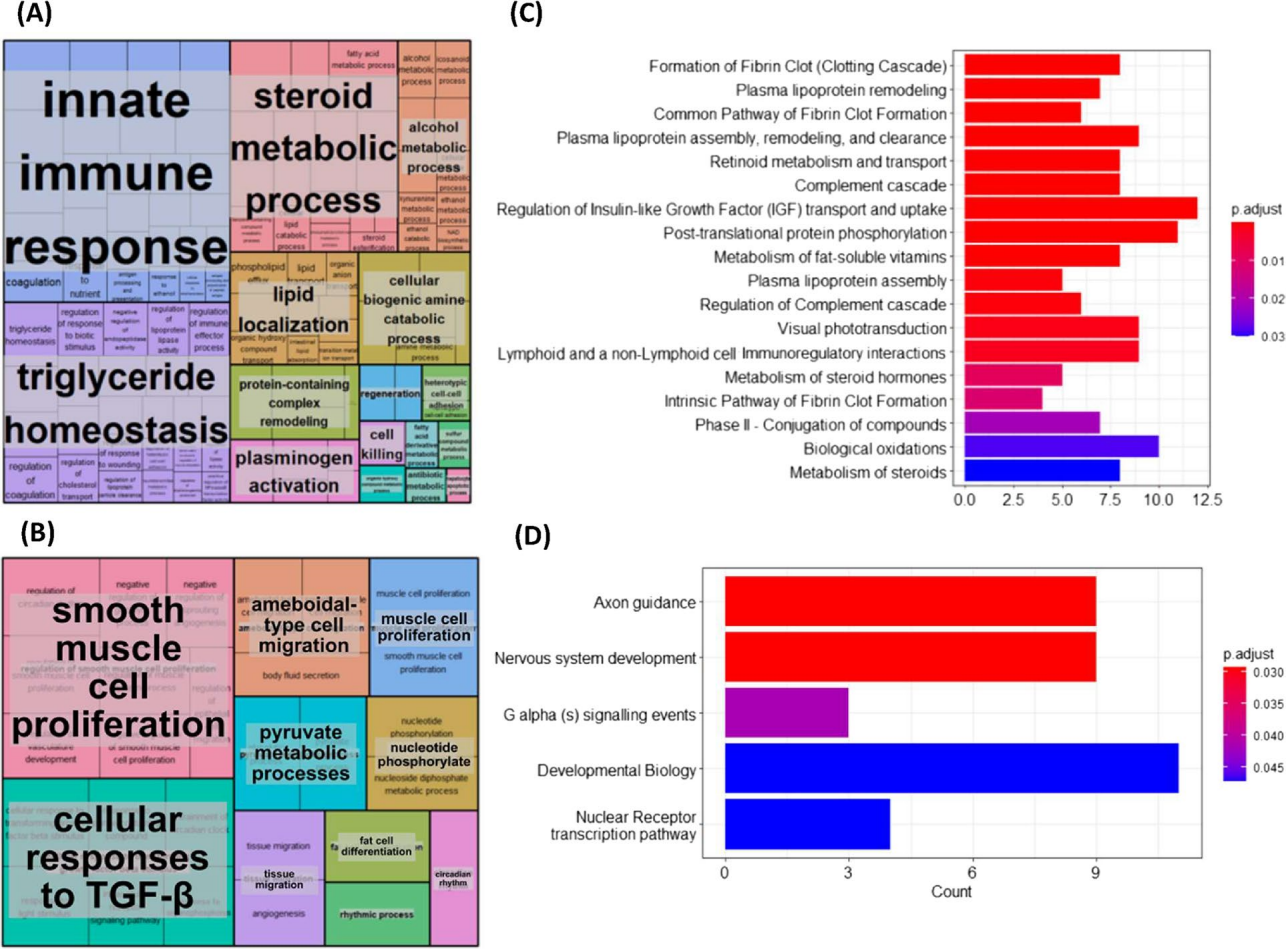
Gene Ontology (GO) and Reactome pathway analysis of genes differentially expressed in aged rat muscle but normalised to youthful expression by caloric restriction (CR). ClusterProfiler (version 3.14.3) package in R (version 4.1), was used to perform GO enrichment analysis. All the genes detected in skeletal muscle by RNA-seq analysis was used as background. A padj < 0.05 was used as the cut off *p*-value. Revigo (http://revigo.irb.hr) then summarised GO terms for genes **A** suppressed by and **B** rescued by CR. Subsequently, the ReactomePA (version 3.15) package in R revealed Reactome pathways significantly (p-adj value =  < 0.05) associated with genes **C** suppressed by and **D** rescued by CR

ClusterProfiler revealed 265 GO terms to be significantly associated with genes suppressed in aged CR rats compared to age rats fed AL. Top terms based on gene size included innate immune response, response to molecule of bacterial origin and complement activation cascade. Revigo summarised genes suppressed by CR to primarily be involved in the innate immune response, triglyceride homeostasis and steroid metabolism (Fig. 3A). Subsequently, genes suppressed by CR were found to be significantly enriched for 18 Reactome pathways, including the clotting cascade, plasma lipoprotein remodelling, plasma lipoprotein assembly, remodelling, and clearance, regulation of IGF transport and uptake by IGF binding proteins, retinoid metabolism and transport, complement cascade and post-translational protein phosphorylation (Fig. 3C).

ClusterProfiler revealed 47 GO terms to be significantly associated with genes rescued in aged CR rats compared to aged rats fed AL. Top terms based on gene size included regulation of circadian rhythm, ATP generation from ADP and muscle cell proliferation. Revigo summarised genes suppressed by CR to primarily be involved in the regulation of smooth muscle cell proliferation, cellular response to transforming growth factor beta, ameboidal-type cell migration, and muscle cell proliferation (Fig. 3B). Subsequently, genes rescued by CR were found to be significantly enriched for 5 Reactome pathways, including axon guidance, nervous system development, G alpha (s) signalling events, developmental biology and nuclear receptor transcription pathway (Fig. 3D).

### Functional and pathway analysis of protein-coding genes differentially expressed uniquely in CR rats

**Fig. 4:**
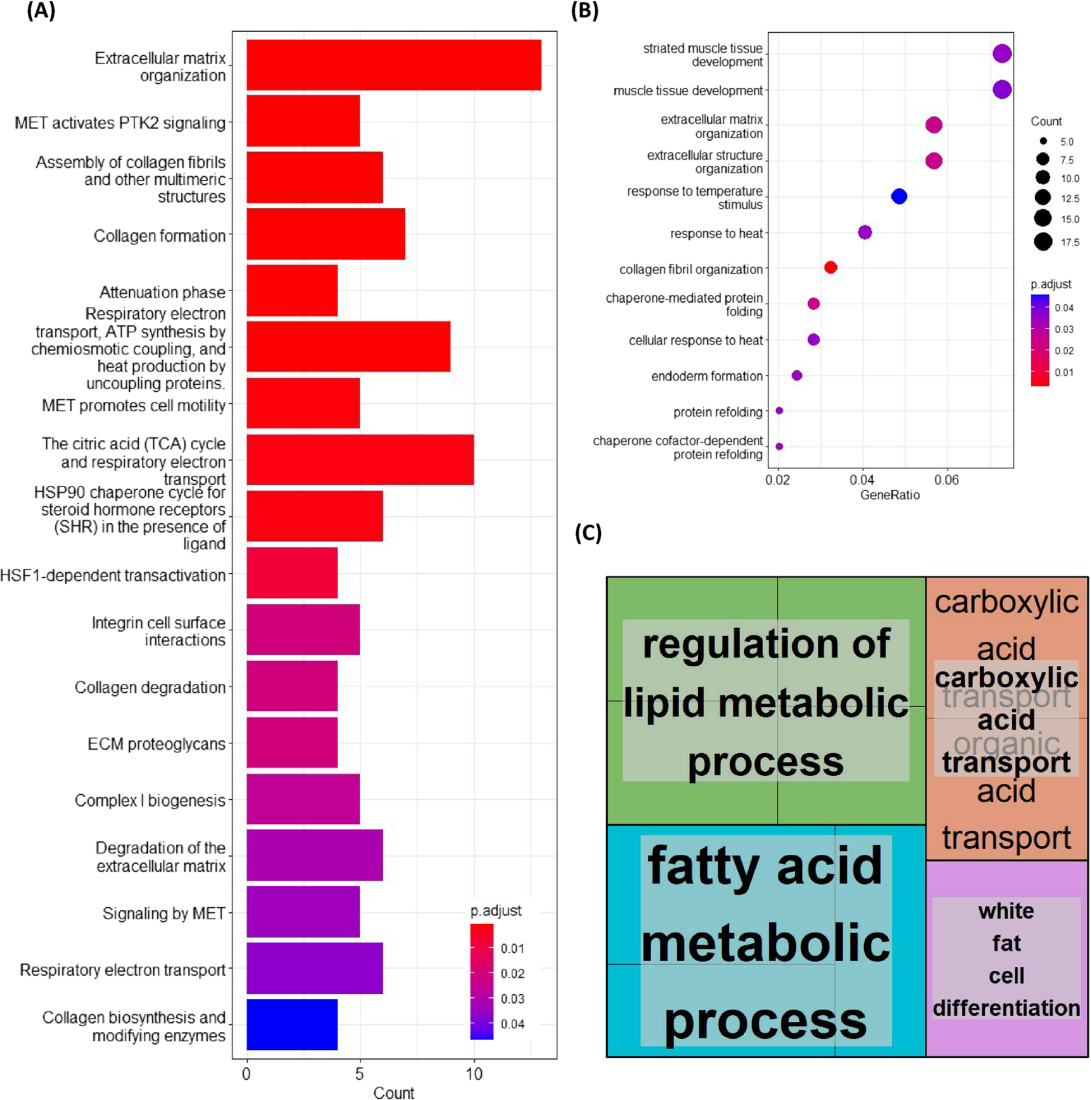
Gene Ontology (GO) and Reactome pathway analysis of genes differentially expressed in the muscle of aged caloric restriction (CR) but not aged non-CR rats. ClusterProfiler (version 3.14.3) and ReactomePA (version 3.15) packages in R (version 4.1) were used to perform GO and Reactome pathway enrichment analyses. All the genes detected in skeletal muscle by RNA-seq analysis was used as background. A padj < 0.05 was used as the cut off p-value. For downregulated genes, significantly associated **A** pathways and **B** GO terms were revealed for uniquely downregulated in old CR. Revigo could not summarise GO terms significantly associated with downregulated genes due to the low number of terms. **C** For upregulated genes, GO terms were revealed, which Revigo (**A**)(**B**)(**C**)(http://revigo.irb.hr) summarised. Only one pathway was found to be over-expressed in CR, which was biological oxidation

ClusterProfiler revealed 14 GO terms to be significantly associated with genes uniquely upregulated in age CR rats. Functional enrichment analysis showed that genes overexpressed under CR were linked to fatty acid metabolic process, white fat cell differentiation, carboxylic acid transport, organic acid transport and regulation of neurotransmitter levels, amongst others GO terms. Revigo summarised these 14 GO terms into 4 categories, these were: regulation of lipid metabolic process, fatty acid metabolic process, carboxylic acid transport and white fat cell differentiation (Fig. 4C). As for pathways, we found one significant term associated with genes that were over-expressed under CR, which was biological oxidations.

ClusterProfiler revealed 12 GO terms to be significantly associated with genes uniquely downregulated by CR. Top terms based on gene size included muscle tissue development, extracellular matrix (ECM) organization, chaperone-mediated protein folding and collagen fibril organization (Fig. 4B). Revigo clustering was not applied given the limited number of terms. Nonetheless, genes uniquely downregulated by CR were found to be significantly enriched for 18 Reactome pathways. These included ECM organization, MET activates PTK2 signalling, the citric acid (TCA) cycle and respiratory electron transport, integrin cell surface interactions and respiratory electron transport (Fig. 4A).

### Functional and pathway analysis of protein-coding genes differentially expressed in ageing but unaffected by CR

**Fig. 5:**
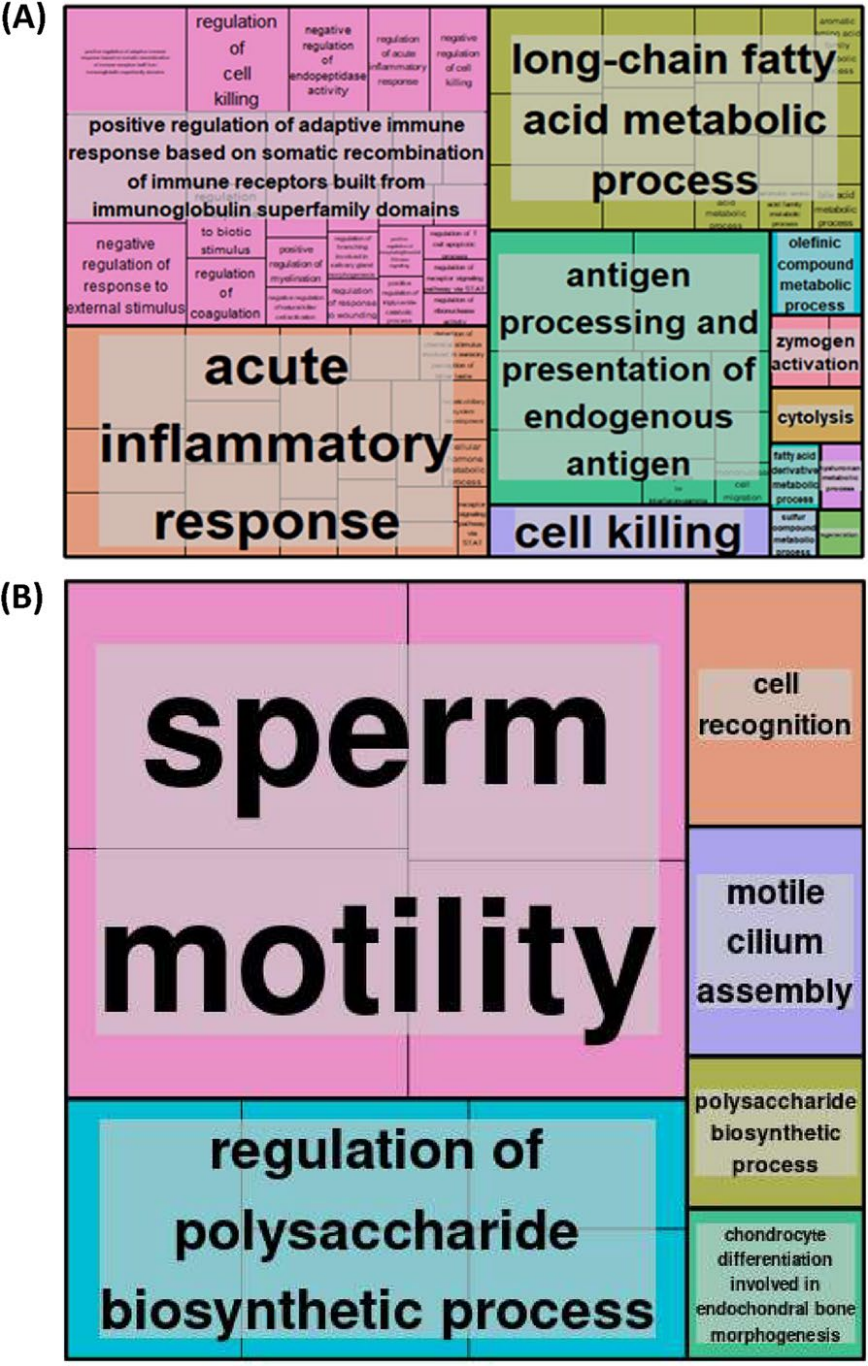
Gene Ontology (GO) analysis of genes differentially expressed in aged rat muscle but unaffected by caloric restriction (CR). ClusterProfiler (version 3.14.3) package in R (version 4.1), was used to perform GO enrichment analysis. All the genes detected in skeletal muscle by RNA-seq analysis was used as background. A padj < 0.05 was used as the cut off *p*-value. Revigo (http://revigo.irb.hr) then summarised GO terms for **A** upregulated genes and **B** downregulated genes

Finally, we investigated the biological processes and the pathways associated with genes commonly upregulated in both the aged muscle of rats fed AL and CR when compared to young rats. ClusterProfiler revealed 254 GO terms to be significantly associated with genes commonly upregulated in aged muscle. Top terms based on gene size included lymphocyte-mediated immunity antigen processing and presentation of peptide antigen via MHC class I, cell killing, leukocyte-mediated immunity, acute inflammatory response and adaptive immune response regeneration. Revigo clustering subsequently summarised these genes to be involved in the positive regulation of adaptive immune response based on somatic recombination of immune receptors built from immunoglobulin superfamily domains, the acute inflammatory response, long chain fatty acid metabolic processes, antigen processing and presenting and cell killing and regeneration (Fig. 5A). Subsequently, upregulated genes in aged rats were found to be significantly enriched for 21 Reactome pathways, including Platelet degranulation, Response to elevated platelet cytosolic Ca2 + , Homeostasis and Regulation of Complement cascade.

ClusterProfiler also revealed 33 GO terms to be significantly associated with genes commonly downregulated in aged muscle. The biological processes most commonly downregulated were found to include sperm motility, cellular processes involved in reproduction in multicellular organisms, cell recognition, and regulation of glucan biosynthetic process. Revigo clustering subsequently summarised these genes to be involved primarily in sperm motility and the regulation of polysaccharide biosynthesis (Fig. 5B). Pathway analysis demonstrated commonly downregulated genes to be associated with 3 significant Reactome pathways. These pathways were the HSP90 chaperone cycle for steroid hormone receptors (SHR) in the presence of ligand, attenuation phase and cellular response to heat stress.

### Weighted gene expression clustering analysis

**Fig. 6:**
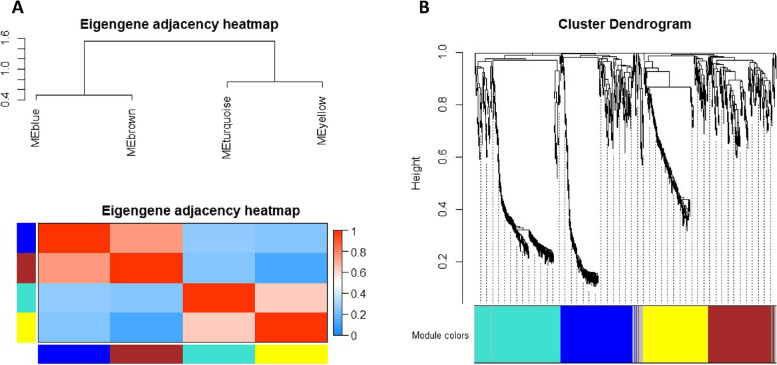
Weighted gene correlation network analysis (WGCNA) for DEG in old rat skeletal muscle. **A** Module eigengene adjacency heatmap was clustered and were merged by a cut-off value of 0.25 and classified as the first principal component of a co-expression module matrix. Heatmap showing the similarity between 4 co-expressed modules (Blue, Brown, Turquoise, and Yellow). Colours indicate the strength of relation between the modules, red is highly associated, and blue shows no relation. **B** Hierarchical clustering of the topological overlap matrix for DEG data between young vs old muscle. Colours at the bottom of the dendrogram denote various clusters, which is detected by the dynamic tree cut algorithm

Weighted Gene Correlation Network Analysis (WGCNA) revealed four co-expression modules (Fig. 6), with two modules, turquoise and yellow, significantly correlated with ageing muscle in old rats. These modules were labelled as ageing-associated due to their relevance to ageing in old rats compared to young ones.

**Fig. 7:**
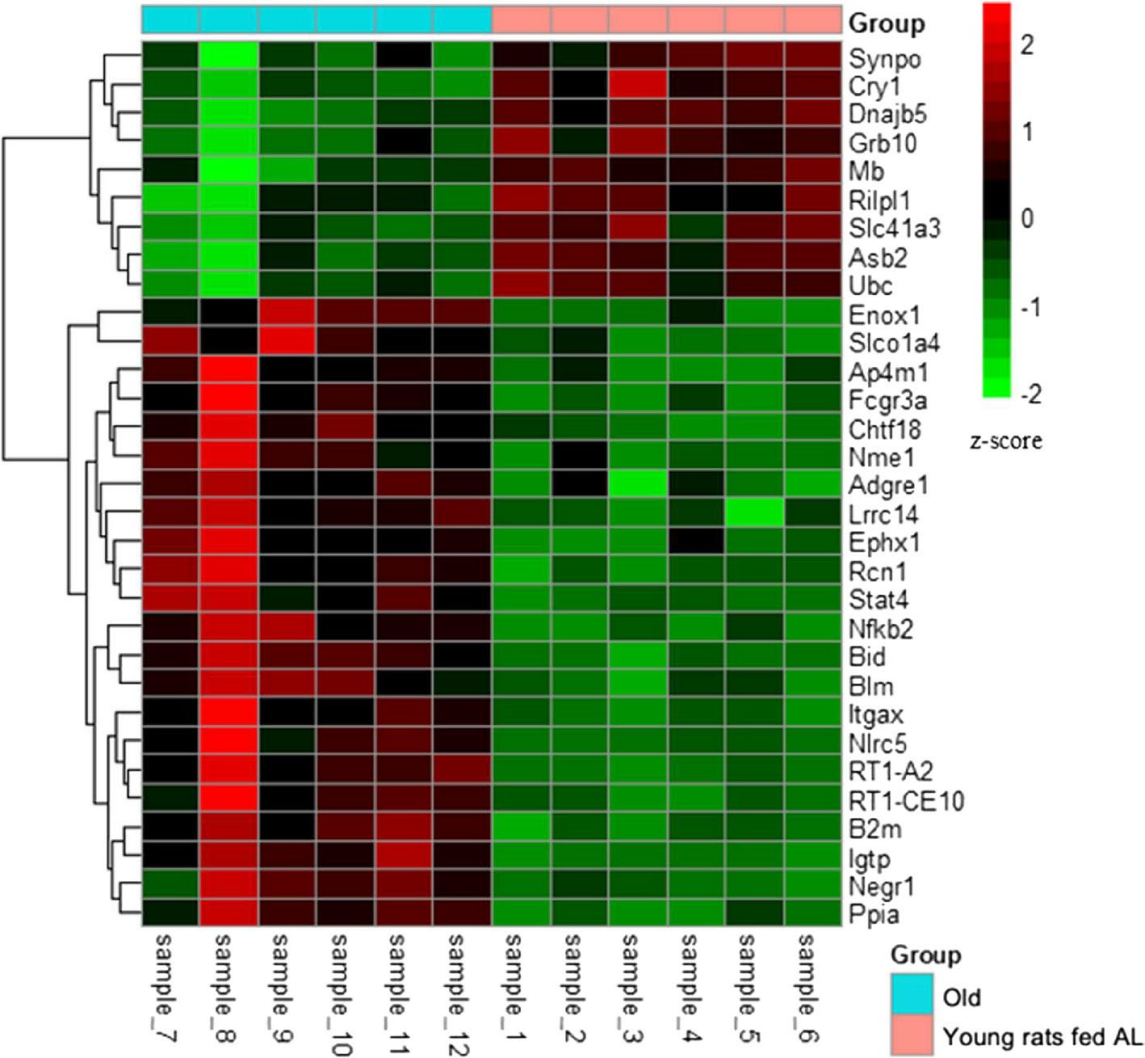
Heatmaps represent the expression level of hub genes from turquoise and yellow module (Age-associated hub genes). Pheatmap package (version 1.0.12) was used to create the heatmap. Normalised read counts of hub genes were used and z-score were computed to produce the heatmap. Red colours represent higher expression and green represent lower expression. X-axis showing samples and y- axis showing genes. Group colour turquoise represents old skeletal muscle, and pink represents young skeletal muscle of rat

Further analysis focused on assessing the association between module membership (MM) and gene significance (GS) for ageing muscle. Genes within the turquoise and yellow modules were considered hub genes if they met stringent criteria (GS > 0.80 and MM > 0.80). Ultimately, two hub genes,

Enox1 and Slco1a4, were identified from the turquoise module, while 29 hub genes, including *Nlrc5, Bid*, *Nfkb2*, *Blm* and *Slc41a3*, were identified from the yellow module (Table 4). Among these hub genes, nine were upregulated and 22 were downregulated in ageing muscle compared to young muscle (Fig. 7).

**Table 4:** Ageing-associated hub genes from turquoise and yellow modules. Weighted gene correlation network analysis revealed several hub genes, defined as any gene with a gene significance value > 0.80 and module membership value > 0.80. **A** Shows hub genes from the turquoise module. **B** Shows hub genes from the yellow module

**Gene**	**log**_**2**_**(FoldChange)**	***p*-value**	**padj**
**A**			
Enox1	0.957	1.16E-05	0.000842
Slco1a4	1.055	1.52E-07	2.74E-05
**B**			
Fcgr3a	2.243	5.59E-07	7.82E-05
Nlrc5	2.103	3.19E-12	3.35E-09
RT1-A2	1.562	3.33E-16	1.12E-12
Itgax	1.454	2.84E-09	8.99E-07
B2m	1.266	4.57E-13	7.68E-10
Bid	1.164	2.76E-12	3.09E-09
Negr1	1.134	1.43E-05	0.000994
RT1-CE10	1.129	4.40E-09	1.34E-06
Igtp	1.010	8.94E-15	2.14E-11
Adgre1	1.003	0.000356	0.011089
Ephx1	0.979	2.14E-06	0.000225
Chtf18	0.896	0.000397	0.011956
Nfkb2	0.886	7.68E-10	2.93E-07
Stat4	0.730	2.23E-07	3.46E-05
Blm	0.703	5.63E-05	0.002862
Nme1	0.696	2.41E-05	0.001445
Lrrc14	0.650	9.65E-05	0.004389
Ap4m1	0.624	0.000136	0.005508
Rcn1	0.599	5.36E-07	7.62E-05
Ppia	0.594	4.84E-07	7.13E-05
Synpo	−0.695	0.000189	0.007002
Grb10	−0.708	0.000122	0.00507
Rilpl1	−0.734	0.000463	0.013262
Cry1	−0.752	7.20E-08	1.47E-05
Mb	−0.762	0.000302	0.009895
Ubc	−0.877	6.59E-05	0.003233
Dnajb5	−0.938	8.59E-06	0.000656
Asb2	−0.961	4.64E-05	0.002493
Slc41a3	−1.004	1.01E-06	0.00013

### Hub genes for yellow and blue modules

**Fig. 8:**
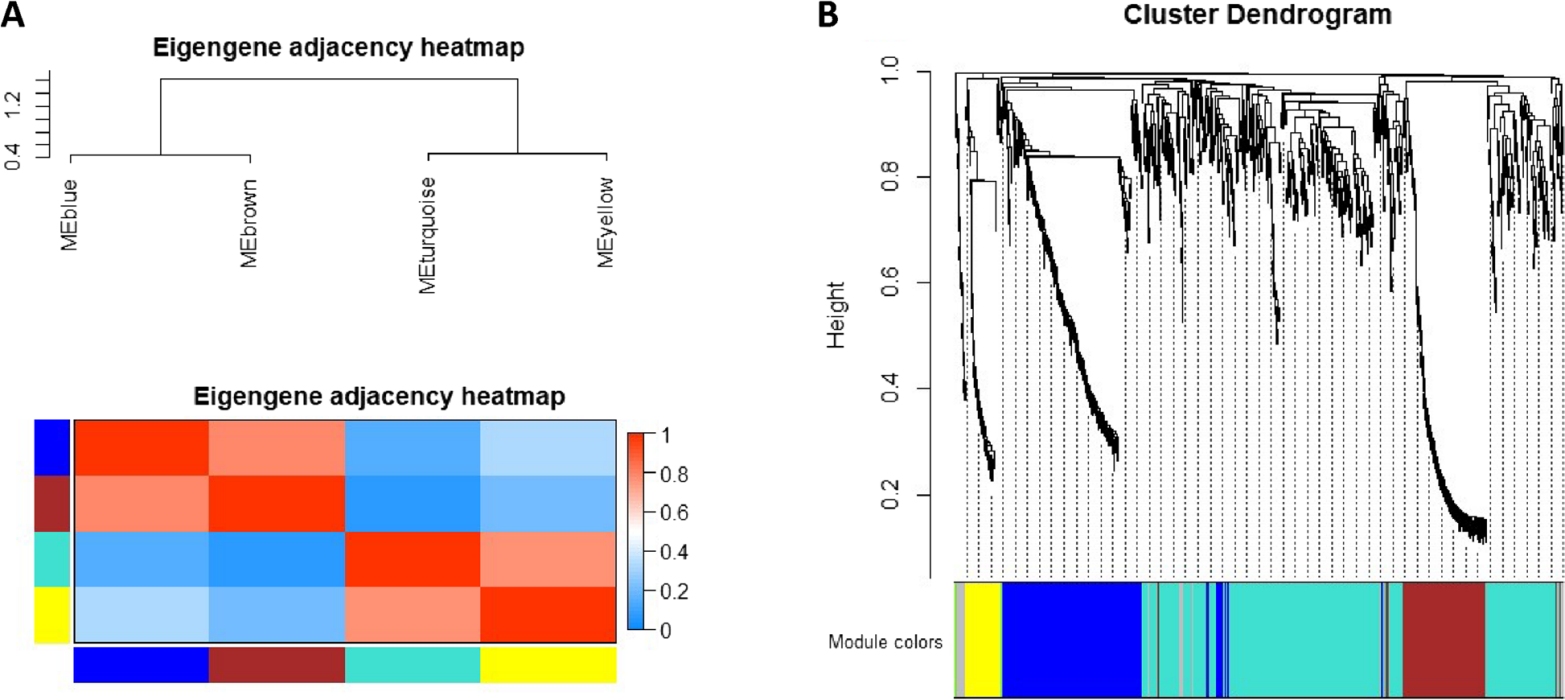
Weighted gene correlation network analysis (WGCNA) of DEG in old CR skeletal muscle from rat. **A** Module eigengene adjacency heatmap was clustered and were merged by a cut-off value of 0.25 and classified as the first principal component of a co-expression module matrix. Heatmap showing the similarity between 4 co-expressed modules (Blue, Brown, Turquoise, and Yellow). Colours indicate the strength of relation between the modules, red is highly associated, and blue shows no relation. **B** Hierarchical clustering of the topological overlap matrix for DEG data between young vs old CR muscle. Colours at the bottom of the dendrogram denote various clusters, which is detected by the dynamic tree cut algorithm

**Fig. 9:**
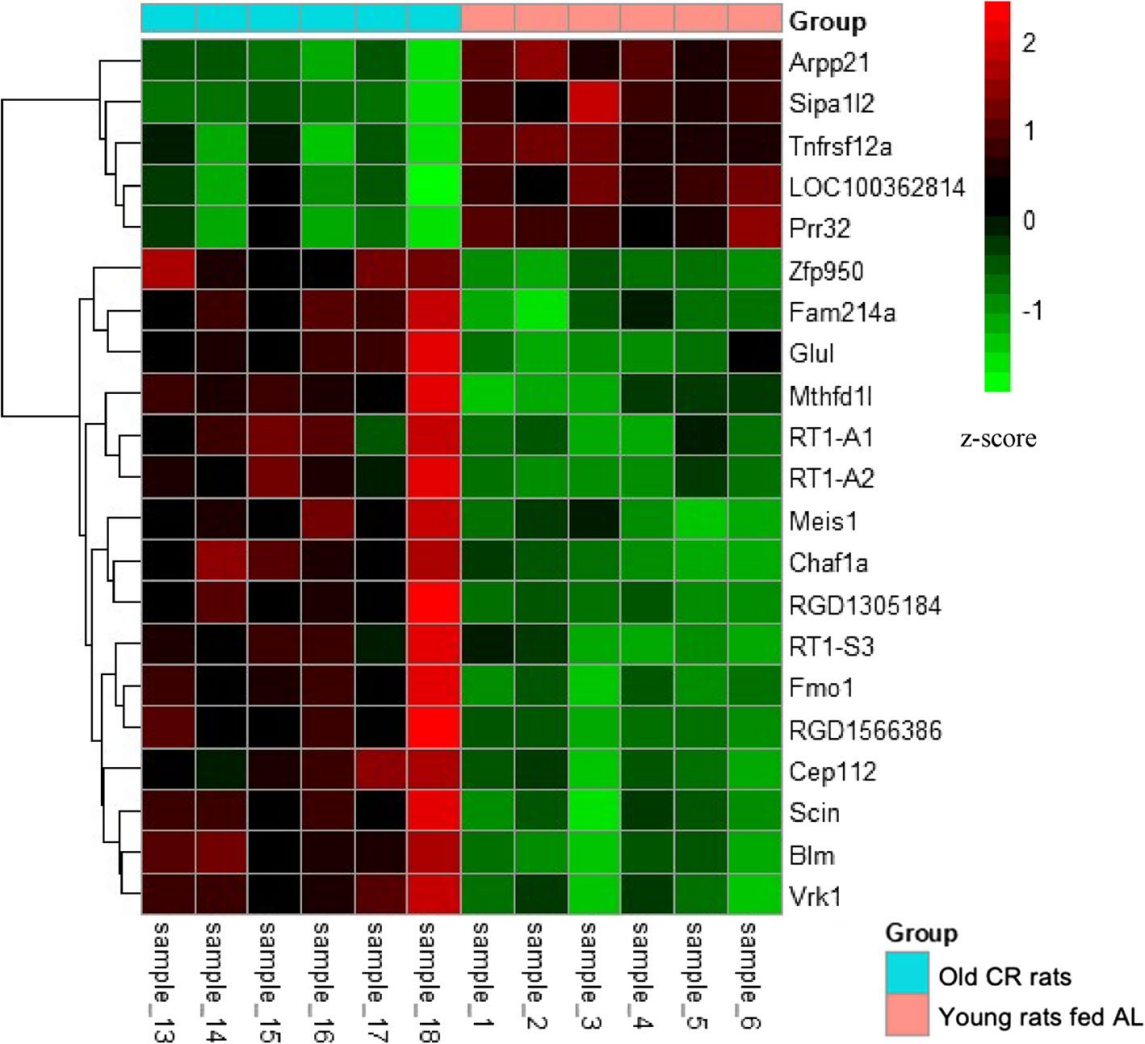
Heatmaps represent the expression level of hub genes from yellow and blue module (CR- associated hub genes). Pheatmap package (version 1.0.12) was used to create the heatmap. Red colours represent higher expression and green represent lower expression. X-axis showing samples and y- axis showing genes. Group colour turquoise represents old CR skeletal muscle and pink represents young skeletal muscle of rat. Sample 1–6 represent 6-month-old and sample 13–18 represent 28 months CR

Using the same approach as above, WGCNA revealed four co-expression modules (Fig. 8) which was then used to identify the CR associated hub genes. WGCNA identified one hub gene, *Zfp950*, from the yellow module and 20 hub genes from the blue module. The combined hub gene set from both modules is presented in a table (Table 5). Examining the impact of muscle ageing under CR, we observed that among the identified hub genes, five were downregulated, including *Arpp21, LOC100362814, Prr32, Sipa1l2* and *Tnfrsf12a*. Meanwhile, 16 genes, including *Blm, Cep112, Fam214a* and *Zfp950*, were upregulated. These findings shed light on specific genes affected by the ageing process under calorie restriction in muscle tissue (Fig. 9).

**Table 5:** CR-associated hub genes from blue and yellow modules. Weighted gene correlation network analysis revealed several hub genes, defined as any gene with a gene significance value > 0.80 and module membership value > 0.80

Gene	log_2_(FoldChange)	*p*value	padj
Arpp21	−1.345	1.23E-08	2.19E-06
Blm	0.770	4.17E-08	6.20E-06
Cep112	0.692	5.64E-05	0.00228
Chaf1a	0.765	6.89E-06	0.000481
Fam214a	0.841	3.39E-07	3.70E-05
Fmo1	1.335	6.96E-10	1.85E-07
Glul	0.700	2.81E-06	0.00023
LOC100362814	−0.610	0.000106	0.003563
Meis1	0.612	0.000109	0.003652
Mthfd1l	0.867	2.74E-05	0.001299
Prr32	−0.779	1.97E-05	0.001017
RGD1305184	2.595	5.12E-07	5.36E-05
RGD1566386	0.591	7.38E-05	0.002769
RT1-A1	0.655	6.16E-06	0.000439
RT1-A2	1.058	8.29E-10	2.06E-07
RT1-S3	1.008	4.52E-07	4.82E-05
Scin	1.130	2.40E-05	0.001183
Sipa1l2	−0.994	3.03E-10	8.93E-08
Tnfrsf12a	−0.872	5.00E-06	0.000367
Vrk1	0.600	2.46E-06	0.00021
Zfp950	0.626	1.01E-07	1.31E-05

### PPI network analysis of ageing-associated and CR-associated differentially expressed genes in aged muscle

**Fig. 10:**
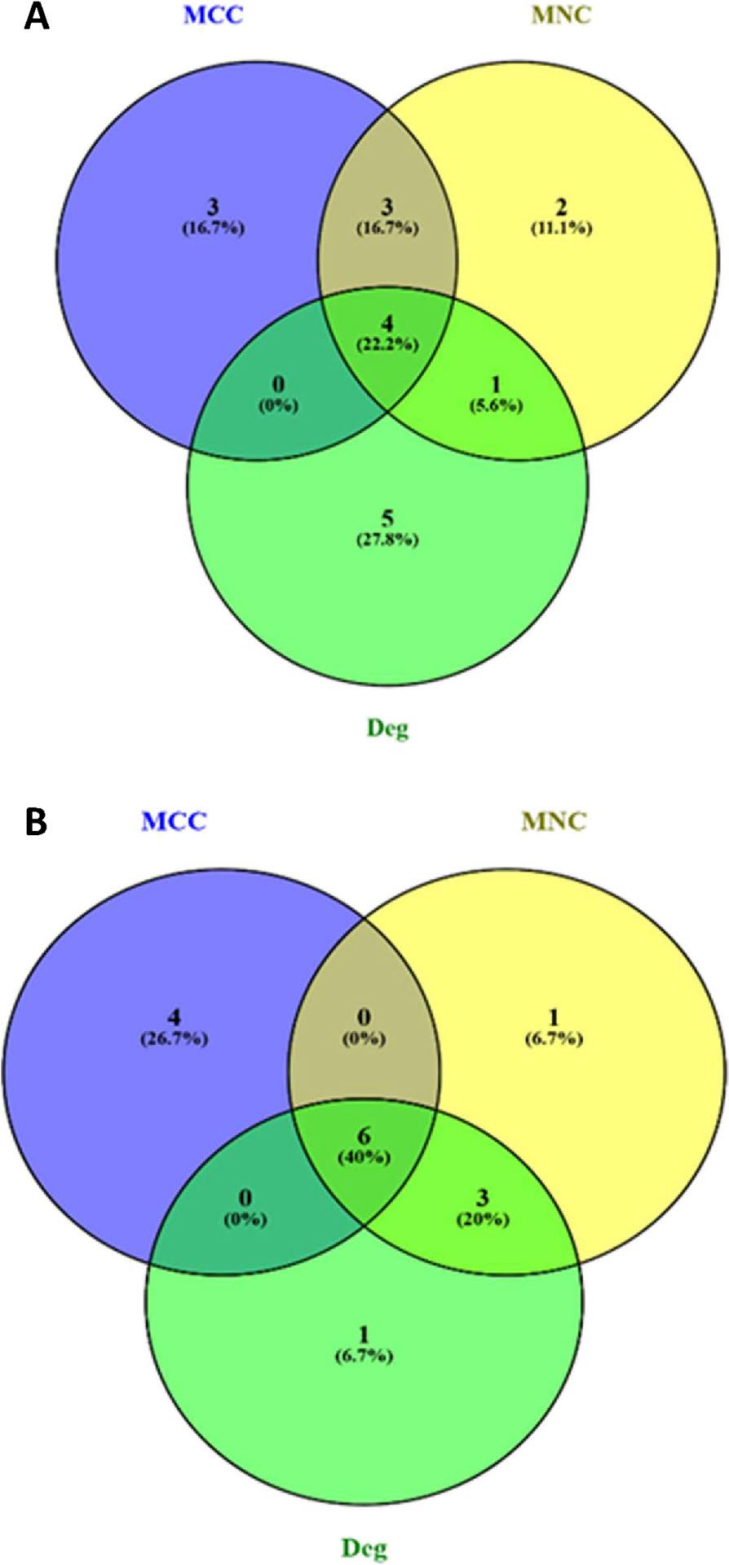
Venn diagrams showing the degree of overlap between results using various method to identify common age associated hub protein from PPI analysis. **A** Yellow module and **B** Turquoise module. Venn diagram was created using “ https://bioinfogp.cnb.csic.es/tools/venny. Hub genes were identified using cytoHubba via 3 different methods: Maximal Clique Centrality (MCC), Maximum Neighbourhood Component (MNC) and the Degree method (Deg). Hub genes identified by more than one method were assumed to be the least likely to be false-positive

To explore ageing-related genes in muscle, PPIs were investigated using the STRING database, which facilitated the identification of known and predicted interaction between proteins for the genes of interest. The yellow module exhibited 172 nodes and 181 edges in its interaction networks, while the turquoise module contained 232 nodes and 665 edges. Key proteins within these networks were identified. In the yellow module, four proteins—*Ifit3, Irf7, Rsad2* and *Usp18*—stood out across different methods, suggesting their significance in muscle ageing (Fig. 10A, Table 6A). These proteins were labelled as ageing-related hub genes, all showing upregulation compared to younger samples. Interestingly, *B2M*, a gene well-characterised to display age-associated dysregulation, appeared both in the PPI network analysis and the WGCNA method. In the turquoise module, six proteins—*Fgg, Fga, Plg, Gc, Serpinc1* and *Fgb*—emerged as prominent hub genes associated with ageing (Fig. 10B, Table 6B). Notably, all six showed upregulation in ageing muscle. However, when comparing these hub genes to the WGCNA method, no overlapping genes were found, indicating distinct findings.

**Table 6:** Ageing-associated protein hubs in rat muscle. Results from CytoHubba analysis for (A) the yellow module and (B) the turquoise module. CytoHubba plugin of Cytoscape (version 3.9.1) was used to rank the nodes by their network features. Top 10 ranked nodes were obtained using MCC, MNC and Deg method from CytoHubba

**MCC method**		**MNC method**		**Deg method**	
Gene.name	Rank	Gene.name	Rank	Gene.name	Rank
**A**					
Ifit3	1	B2m	1	Irf7	1
Irf7	2	Parp14	2	B2m	2
Usp18	3	Irf7	3	Psmb8	3
Rsad2	4	Ifit3	3	usp18	4
Parp14	5	Usp18	3	IL2RB	5
Oasl2	6	Cd8b	6	Cd28	6
Rtp4	7	Rsad2	6	Ifit3	7
Ifi44	8	Oasl2	8	Rsad2	8
Dhx58	9	Ifi44	9	Itgb2	9
Irf9	10	Klrc1	10	Gbp2	10
**B**					
Fgg	1	Serpinc1	1	Serpinc1	1
Fga	1	Fgg	1	Fgg	1
Plg	3	Fga	1	Fga	1
Gc	4	Plg	4	Plg	4
Serpinc1	5	Gc	5	Alb	5
Fgb	6	Apob	6	Gc	6
Cyp1a2	7	Alb	7	Apob	7
Cyp2b3	7	Fgb	7	Fgb	8
Cyp2c6v1	9	F2	9	Cpb2	9
Cyp2c22	10	Apoa1	9	F2	9

**Fig. 11:**
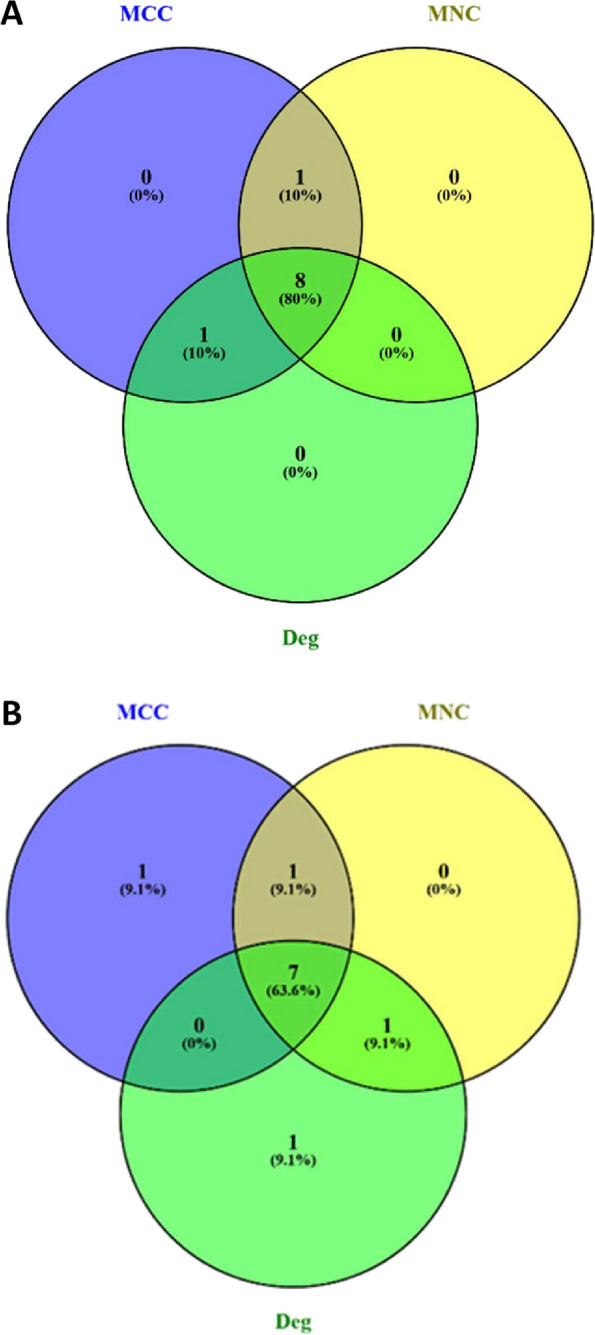
Venn diagram showing over-lapping of hub protein from different methods for old CR skeletal muscle to identify common hub protein (from PPI analysis). **A** Yellow module **B** Blue module. Venn diagram was created using “ https://bioinfogp.cnb.csic.es/tools/venny. Hub genes were identified using cytoHubba via 3 different methods: Maximal Clique Centrality (MCC), Maximum Neighbourhood Component (MNC) and the Degree method (Deg). Hub genes identified by more than one method were assumed to be the least likely to be false-positive

To explore genes associated with muscle ageing in CR rats, PPIs were investigated using the STRING database. The yellow module exhibited 51 nodes with 72 edges in its interaction network, while the blue module contained 224 nodes and 157 edges. Key proteins within these networks were identified. In the yellow module, eight hub genes emerged as significant across the methods—*GC, Alb, Apoa1, Ambp, Plg, F2, Apoh* and *Orm1* (Fig. 11A, Table 7A). Similarly, all these hub genes displayed upregulation in the aged muscle of CR rats when compared to the muscle of young rats fed AL. In the blue module, seven proteins—*Irf7, Ifit3, Usp18, Mx1, Rsad2*, *Oasl2* and *Rtp4*—were significant across all three methods (Fig. 11B, Table 7B). These proteins all exhibited upregulation in the aged muscle of CR rats when compared to the muscle of young rats fed AL. Notably, however, no common hub genes were found between the CytoHubba methods and the WGCNA (MM vs GS).

**Table 7:** CR-associated protein hubs in aged rat muscle. Results from CytoHubba analysis for (A) the yellow module and (B) the blue module. CytoHubba plugin of Cytoscape (version 3.9.1) was used to rank the nodes by their network features. Top 10 ranked nodes were obtained using MCC, MNC and Deg method from CytoHubba

**MCC method**		**MNC method**		**Deg method**	
Gene.name	Rank	Gene.name	Rank	Gene.name	Rank
**A**					
Gc	1	Gc	1	Alb	1
Alb	2	Alb	2	Gc	2
Apoa1	3	Plg	2	Ambp	3
Ambp	4	Ambp	4	Plg	3
Plg	5	F2	5	Apoa1	5
F2	6	Apoa1	5	F2	6
Apoh	7	Apoh	7	Apoh	7
Orm1	8	Orm1	7	Orm1	7
Hp	8	Hp	7	Hrg	7
Hrg	10	Apoh	7	Hp	7
**B**					
Irf7	1	Irf7	1	Irf7	1
Ifit3	2	Ifit3	2	Ifit3	2
Usp18	3	Mx1	3	Mx1	3
Mx1	4	Usp18	3	Usp18	3
Rsad2	5	Mx1	5	Mx1	5
Oasl2	6	Rsad2	5	Rsad2	5
Rtp4	7	Oasl2	7	Oasl2	7
Mx1	8	Parp14	8	Cd8a	7
Dhx58	9	Rtp4	8	Parp14	9
Ifi44l	10	Dhx58	10	Rtp4	9

### Analysis of overlap between ageing-associated and CR-associated hub genes

**Fig. 12:**
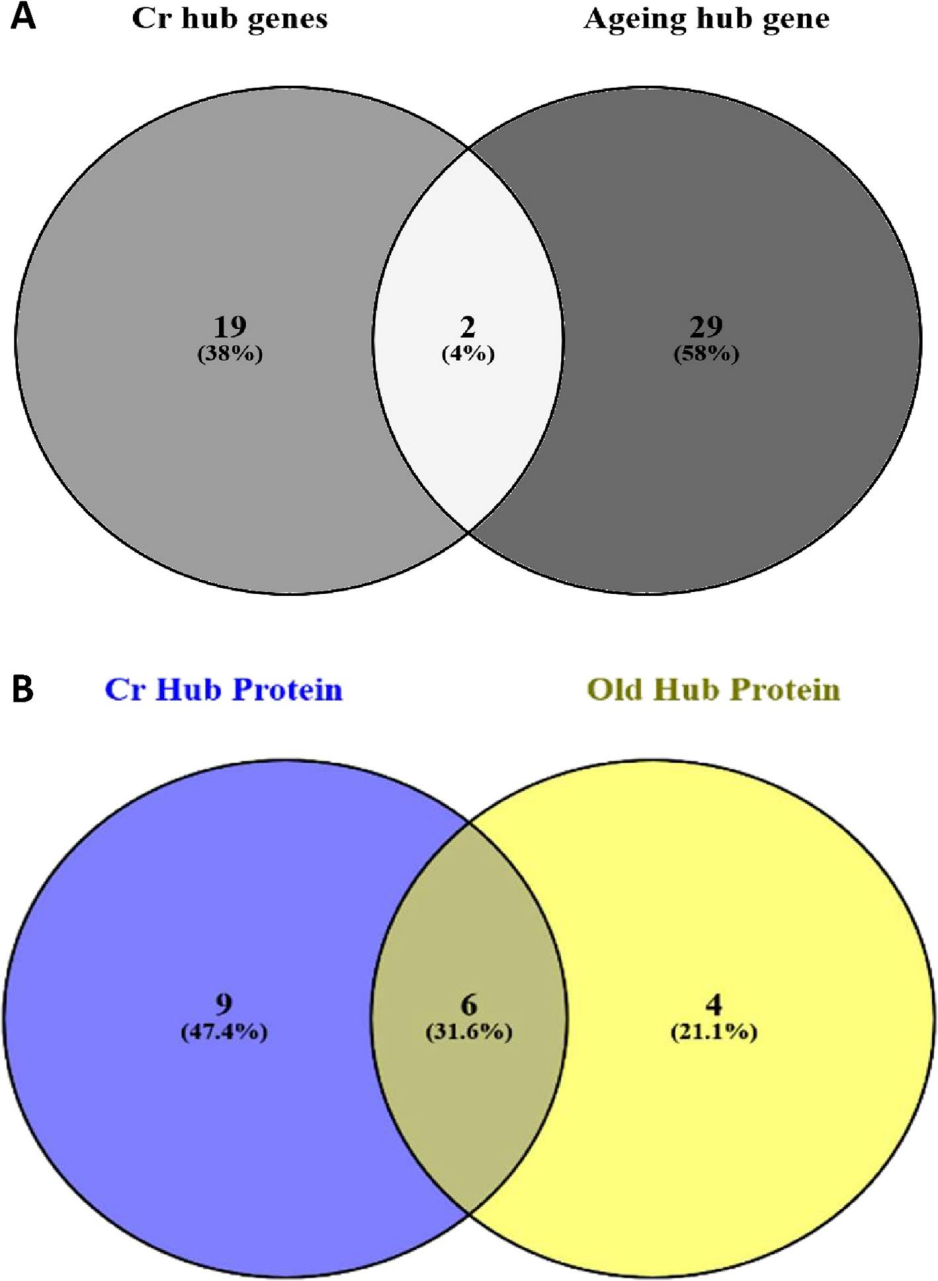
Venn diagram showing over-lapping of hub genes from WGCNA and PPI method. Venn diagram was created using “ https://bioinfogp.cnb.csic.es/tools/venny/”. **A**. Showing common hub genes between old skeletal muscle and old CR skeletal muscle identified using WGCNA MM vs GS method. **B**. Showing overlapping of hub proteins identified using PPI from old CR skeletal muscle to old skeletal muscle

The WGCNA method found only 2 hub genes to be affected by both ageing and CR, *Blm* and *RT1-A2 (*Fig. 12A). However, PPI methods identified 6 hub genes to be affected by both ageing and CR (Fig. 12B, Table 8). These included *Gc, Plg, Irf7, Ifit3, Usp18* and *Rsad2*. Hub genes unique to old CR muscle included *Alb, Apoa1, Ambp, F2, Apoh, Orm1, Mx1, Oasl2* and *Rtp4*, meanwhile, hub genes unique to old muscle included *Fgg, Fga, Serpinc1* and *Fgb*.

**Table 8:** The overlap of hub genes identified by protein–protein interaction PPI network methods between the muscle of aged rats fed ad libitum and aged rats caloric restricted (CR). Eight out of twelve hub genes involved in ageing were also involved in responses to CR. This suggests an incomplete reversal of the ageing phenotype by CR. Nine hub genes were found to mediate the unique effects of CR, independent of ageing reversal

Hub Genes Unique to old CR Muscle		Hub Genes Unique to Aged AL Muscle		Hub Genes Common to Both CR and Ageing	
Symbol	Gene Description	Symbol	Gene Description	Symbol	Gene Description
Alb	Albumin	Fgg	Fibrinogen gamma	Gc	Vitamin D binding protein
Apoa1	Apolipoprotein A-I	Fga	Fibrinogen alpha	Plg	Plasminogen
Ambp	Alpha-1-microglobulin	Fgb	Fibrinogen beta	Irf7	Interferon regulatory factor 7
F2	Coagulation factor II	Serpinc1	Antithrombin	Ifit3	Interferon Induced Protein With Tetratricopeptide Repeats 3
Apoh	Apolipoprotein H			Usp18	Ubiquitin-specific protease 18
Orm1	Alpha-1-acid glycoprotein 1			Rsad2	Rsad2
Mx1	Interferon-induced GTP-binding protein Mx1			Blm	BLM RecQ Like helicase
Oasl2	2'−5' oligoadenylate synthetase-like 2			RT1-A2	Mature alpha chain of MHC class I antigen
Rtp4	Receptor transporter protein 4				

## Validation of RNA-seq analysis using qRT-PCR

**Fig. 13:**
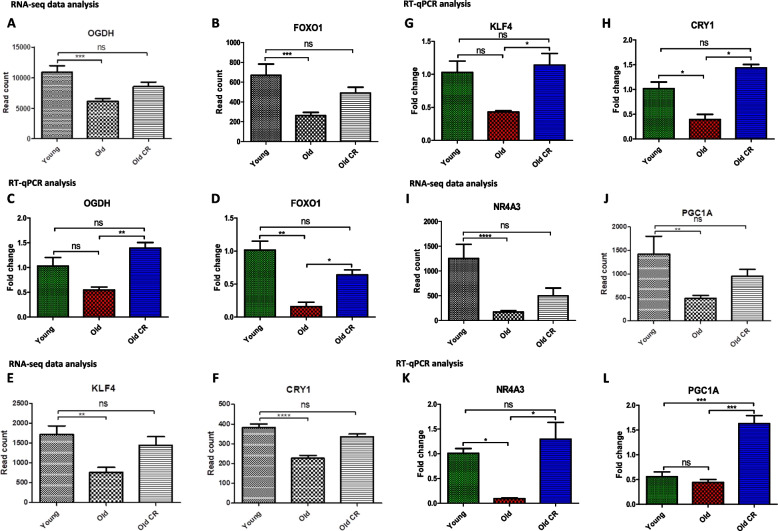
Normalised read count from RNA-seq data compared to relative expression levels in fold change from RT-QPCR. For rt-qpcr samples were calculated relative to Fbxo45 mRNA levels and were compared using One-Way ANOVA test. Of the 6 RNAs selected for analysis, statistically significant changes in expression with old and old-CR samples compared to young were identified for OGDH, FOXO1, KLF4, CRY1, NR4A3 and PGC1A mRNAs (**A**-**L**), *p*- values denoted by a star asterisk. All *p*-values are unadjusted for rt-qpcr, all *p*-values are adjusted for RNA-seq analysis. Error bars represent mean ± SEM

RNA-seq results suggested all 6 of the genes selected for validation to be significantly downregulated in the aged AL-fed rats when compared to the young AL-fed rats, and to be significantly upregulated in the aged CR rats when compared to the aged, AL-fed rats. On the other hand, RT-qPCR found only 4 out of 6 to be significantly downregulated [(*FOXO1*, *p* = 0.002), (*KLF4*, *p* = 0.02), (*CRY1*, *p* = 0.001), (*NR4A3*, *p* = 0.004)] in the aged, AL-fed rats when compared with young AL-fed rats (Fig. 13). *OGDH* and *PGC1A* were not significantly downregulated in old samples according to the RT-qPCR analysis, potentially due to limited sample size (*n* = 3). Expression of *OGDH* was higher in the aged rats fed AL, but not significantly so (Fig. 13). Nonetheless, consistent with RNA-seq results, RT-qPCR analysis suggested all 6 selected genes to be upregulated/rescued in the aged CR rats when compared to the aged, AL-fed rats.

## Discussion

### Differentially expressed genes

As expected, we found many genes to be differentially expressed between the muscle of aged and youthful rats fed AL. Furthermore, CR was found to suppress 69.7% and rescue 57.8% of the genes found to be upregulated and downregulated in aged muscle, respectively. Our work aligns with a previous study on CR, which demonstrated that CR induces changes in the skeletal muscle transcriptional profile in humans and rats, resembling those seen in younger individuals [38]. In addition, our previous study found CR to reverse differential expression of a similar percentage of transcripts in the brain of these animals [39]. CR reversed most of the age-related changes in transcriptomic profiles.

We briefly compared our findings with several previous studies to identify common DEGs related to muscle ageing. A recent RNA-seq study [17] identified 506 significantly dysregulated RNAs in ageing human muscle, with only 13(1.59%) overlapping DEGs in our study, including *Cpeb2, Chrna1, Fgf7, Mylk4, Gbp2, Tet2, Sln, Irs2, Ostn*, and *Tmem158*. Another study [40] identified 596 DEGs in ageing human muscle via microarray, of which 13 (1.59% relative to our study) overlapped with our findings, including *Ppargc1a, Irs1, Cebpb,* and *Cdkn2b*. Similarly, comparing to a mouse study on muscle ageing [15], we found 28 common DEGs (3.42%). Comparing our results with a single-cell RNA-seq study in *Rattus norvegicus* [41], we found 86 common DEGs (10.5%). Comparing to another similar study [14] we found 185 overlapping DEGs (22.56%), likely due to the similar species used. Comparing our study with a meta-analysis on ageing transcriptomes [42], we found 60 overlapping genes (7.33%). In the Human Ageing Genomic Resources (HAGR) GenAge human database [43], only 20 DEGs overlapped (2.56%) (Additional file 1, Figure S1).

We also briefly compared our CR responsive genes with previous studies. For instance, 9 (1%) DEGs overlapped with a CR study conducted in human muscle [44]. Despite limited overlap, likely due to species differences and varying *p*-value criteria, approximately 14 to 299 (1.58–33.67%) of the DEGs in our study overlapped with other CR studies [14, 16, 23] (Additional file 1, Figure S1).

We identified the top ten most significantly dysregulated genes in ageing muscle based on adjusted *p*-values. Six upregulated genes (*Dclk1, Igtp, Lgals1, B2m, Ctxn3, and Nlrc5*) and five downregulated genes *(Kcnc1, Nr4a3, Col1a1, Maf, and Ddit4*) were also reported in previous studies [14, 41, 42]. This consistency suggests these genes play crucial roles in skeletal muscle ageing and might contribute to sarcopenia.

We hypothesise the modest overlap in DEGs across studies may result from species differences, varied methodologies (microarray vs. RNA-seq), small sample sizes, and biological variability. Differences in age ranges and health conditions of study cohorts may also influence molecular signatures of ageing. Further research with larger, more diverse cohorts and multi-platform integration is needed to discern conserved from species-specific transcriptional changes in aged muscle. Our findings contribute to the growing understanding of muscle ageing mechanisms.

GO and pathway enrichment analysis of differentially expressed genes replicated the well-characterised finding that ageing regardless of tissue type is generally characterised by immune/stress response gene over-expression and metabolic/developmental gene under-expression [42]. Indeed, the genes most significantly upregulated in aged rats fed AL were associated with protein folding and immune responses, corroborating influential theories of ageing [45, 46]. CR suppressed many of the genes associated with these processes, normalising the aged phenotype. Interestingly, consistent with programmatic accounts of ageing [47], the genes that were most significantly downregulated in aged rats fed AL were genes related to developmental biology. These genes were rescued by CR.

While CR did normalise many biological pathways perturbed in ageing, it also uniquely altered pathways that are perhaps crucial for normal muscle function. Genes upregulated uniquely by CR in aged rats were most associated with fatty acid metabolism, meanwhile, those downregulated uniquely by CR rats were most associated with ECM organisation and cellular respiration. Although several of these changes are likely involved in the metabolic shift towards increased fat utilisation with CR [48], effects on the ECM may contribute to unwanted side-effects. Indeed, reduced ECM synthesis is thought to contribute to exacerbated age-associated deficits in wound healing [49] and skin quality [50] concomitant with ageing. Saying this, reducing ECM deposition has been shown to contribute to the anti-hypertensive [51] and neuroprotective [52] effects of CR. Ultimately, how CR-mediated alterations in ECM organisation might alter sarcopenia and overall muscle health should be explored further.

### Ageing- and CR-associated hub genes

Several hub genes associated with both ageing and CR responses in rat muscle were identified. The function of these genes and how these might contribute to muscle ageing are listed below.

1. ***Blm*** (a 3′ − 5′ ATP-dependent RecQ DNA helicase) is a necessary genome stabilizer, which controls DNA replication, recombination and repair. Its dysregulation may perturb DNA repair processes within muscle cells, contributing to the accumulation of DNA damage with age [53].
2. ***RT1-A2*** (RT1 class Ia, locus A, sub-locus 2) is involved in presenting antigens to immune cells [54]. Its dysregulation with ageing may contribute to chronic inflammation but its overall role in the ageing process is largely unexplored.
3. ***Gc*** (Vitamin D-binding protein) is responsible for the transport and regulation of vitamin D precursors and metabolites, implicating dysregulated Vitamin D signalling in age-related muscle decline, as has been shown previously [55].
4. ***Plg*** (Plasminogen) is a precursor of the enzyme plasmin, involved in breaking down blood clots. In muscle ageing, its dysregulation could impact repair processes and regeneration efficiency, as has been hypothesised previously [56].
5. ***Irf7*** (Interferon regulatory factor 7) is involved in interferon-mediated immune responses. Its overexpression (although only in adipose tissue) has been found to dysregulate mitochondrial function and amino acid metabolism [57]. Furthermore, *Irf7* has recently been shown to be involved in the modulation of satellite cell proliferation and regeneration with age [58]. These functions possibly contribute to its effects on muscle loss.
6. ***Ifit3*** (Interferon-induced protein with tetratricopeptide repeats 3) and *Usp18* (Ubiquitin-specific protease 18) are also involved in interferon-mediated immune responses. Their dysregulation with ageing may contribute to chronic inflammation phenotypes [59, 60]. *Usp18* is also a known regulator of muscle cell differentiation [60].
7. ***Rsad2***(Radical S-adenosyl methionine domain-containing protein 2) plays a role in defence against viruses. Its involvement in muscle ageing likely relates to its ability to modulate cellular responses to stress, as has been shown in age-related macular degeneration [61, 62].

Furthermore, *Bid* and *B2m* also appeared to be involved both in ageing and in responses to CR. Although neither gene was identified as a CR-related hub gene, they were both strongly upregulated by CR and shown to be ageing-associated hubs genes. *Bid* (BH3 interacting domain death agonist), a pro-apoptotic gene [63], potentially contributes to muscle ageing by accelerating the apoptosis of differentiated and satellite cells. In addition to pro-apoptotic functions, *Bid* has been shown to be involved in metabolism and DNA damage [64]. Conversely, *B2m* (β2-microglobulin), is linked to senescence [65], hinting at a potential role for senescence suppression in CR-mediated muscle ageing protraction. Additionally, *NFkB2* (Nuclear Factor Kappa B Subunit 2) was an ageing-associated hub gene, which is a well-characterised inflammatory regulator and known to be associated with the loss of muscle mass [66].

Some intriguing hub genes uniquely associated with CR, included *ARPP21, VRK1, Glul* and *Orm1*. *ARPP21* is associated with myogenic differentiation [67]. *VRK1* upregulation by CR, potentially affects muscle health positively via downstream AMPK activation [68]. Lastly, dietary intake of the amino acid glutamine is thought to aid in the growth of maintenance of muscle mass but not in the elderly [69]. It is interesting, therefore, that *Glul* (glutamine ammonia ligase) was overexpressed in the muscle of CR rats. *Orm1* (orosomucoid 1), a crucial acute-phase plasma protein, is involved in controlling angiogenesis and regulating inflammation and the immune system [70]. However, its upregulation has also been further shown to enhance glycogen storage in the muscle, as well as increase muscle endurance [71]. This finding suggests CR may also modulate genes that are involve in improving muscle strength and endurance via mechanisms independent of those affected by ageing.

### Limitations

The primary limitations of this study were the small number of biological replicates (*n* = 6) and RNAs the fact that they were collected from skeletal muscle stored over 18 years ago. However, the RNA samples prior to sequencing were assessed and passed the quality and integrity checks (Additional file 1, Figure S2). We employed moderate criteria to screen the genes, in the data cleaning process and most importantly differentially expressed genes were validated using qPCR.

Unfortunately, comparisons with other studies revealed limited overlap between the genes shown to be differentially expressed in aged muscle in our study and those reported in the literature. For example, only 13(1.59%) genes were differentially expressed in aged muscle in both our study and another human study [17], only 13 (1.59%) genes were differentially expressed in aged muscle in both our study and a human study [40]. There was however, a great degree of overlap (185 genes, 22.59%) between the genes identified by our study of rat muscle ageing and a study of mouse muscle ageing [14]. Equally, our results displayed a good degree of overlap (60 genes, 7.33%) of the muscle data in a meta-analysis of tissues-specific transcriptomic correlates of ageing [42].

Despite the limited overlap, genes consistently dysregulated in ageing muscle across various studies, such as *Cepb1*, are likely most strongly involved in muscle ageing and potentially in the development of sarcopenia. Nonetheless, the lack of replication suggests that more extensive investigations involving larger cohorts and diverse populations are necessary to elucidate common versus specific transcriptional changes in ageing muscle. Among the 888 genes differentially expressed in the aged muscle of CR rats when compared to the muscle of aged rats fed AL, 314 were also noted in prior studies across different species, indicating somewhat greater consistency in findings.

## Conclusions

Overall, using RNA-seq data from the muscle of young AL-fed and CR rats and aged AL-fed and CR rats, we identified changes in the expression protein-coding genes consequent to both ageing and CR. Several of the identified genes were previously known to be involved in muscle ageing and responses to CR, replicating previous findings. Indeed, we found inflammatory genes to display a large degree of expression change in muscle ageing and this to be ameliorated by CR. In addition to known genes, our results implied genes involved in circadian rhythm regulation and developmental biology to perhaps play a more important role in muscle ageing than previously realised. Additionally, using co-expression analysis and PPI network analysis, we identified key hub genes associated with these pathways. Knowledge about these key proteins may offer novel insights into the mechanisms via which the muscle ageing process could perhaps be perturbed, without having to implement CR. These key genes should be further studied using in-vitro or in-vivo ageing models. In summary, our work indicates that CR is slowing down, but not completely preventing, age-related muscle changes. Several age-related muscle changes are still detected despite CR, suggesting that there are factors driving these changes that are not modified by CR feeding alone. More research is needed to further unravel these mechanisms. However, the partial protective effect of CR illustrates the modifiable nature of the muscle ageing process.

## Supplementary Information

Additional file 1. Comparing the DEG with previously published studies on muscle ageing or CR (Figure S1) and RNA quality analysis (Figure S2). This file contains a Venn diagram showing the common differentially expressed genes between our study and previous studies on muscle ageing and calorie restriction (Figure S1). In addition, the file also contains quality analysis of RNA samples as observed in 1% agarose gel (Figure S2).

## Data Availability

The raw data can be accessed on GEO accession GSE255551.
