# Supplementary material for: Unravelling the transcriptomic symphony of muscle ageing: key pathways and hub genes altered by ageing and caloric restriction in rat muscle revealed by RNA sequencing
Source: BMC Genomics. 2025 Jan 13;26:29. doi: 10.1186/s12864-024-11051-1 (PMC11727704; doi:10.1186/s12864-024-11051-1)
Supplement: Supplementary file 1 — Additional file 1. Comparing the DEG with previously published studies on muscle ageing or CR (Figure S1) and RNA quality analysis (Figure S2). This file contains a Venn diagram showing the common differentially expressed genes between our study and previous studies on muscle ageing and calorie restriction (Figure S1). In addition, the file also contains quality analysis of RNA samples as observed in 1% agarose gel (Figure S2). [file 12864_2024_11051_MOESM1_ESM.pdf]

Figure S1. Comparing the DEG with previously published studies on muscle ageing or CR.

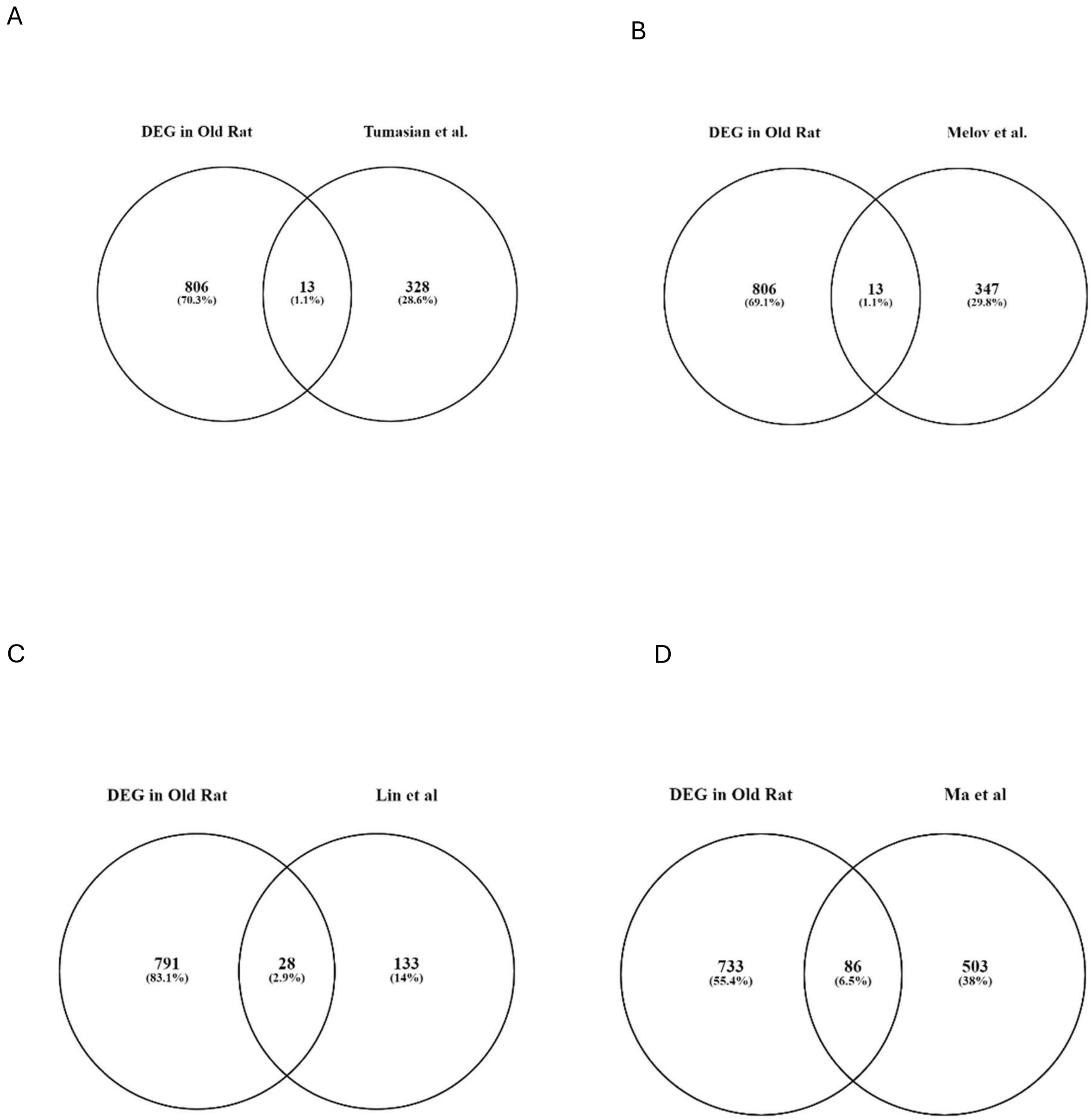

E

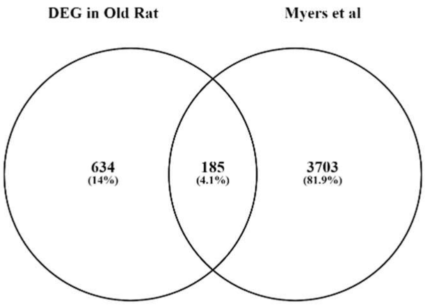
DEG in Old Rat
Myers et al
634
(14%)
185
(4.1%)
3703
(81.9%)

F

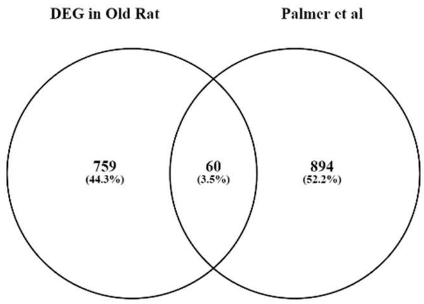
DEG in Old Rat
Palmer et al
759
(44.3%)
60
(3.5%)
894
(52.2%)

G

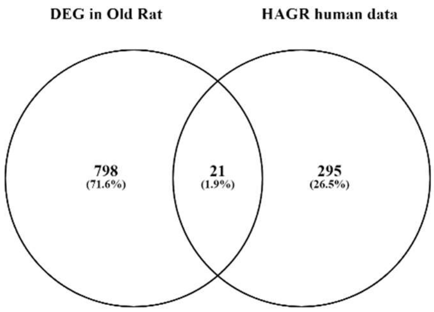
DEG in Old Rat
HAGR human data
798
(71.6%)
21
(1.9%)
295
(26.5%)

H

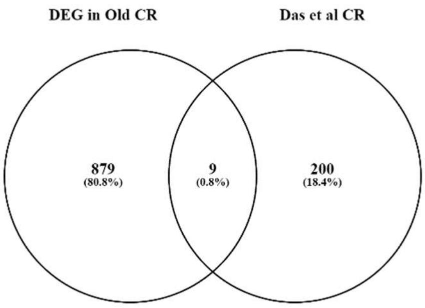
DEG in Old CR
Das et al CR
879
(80.8%)
9
(0.8%)
200
(18.4%)

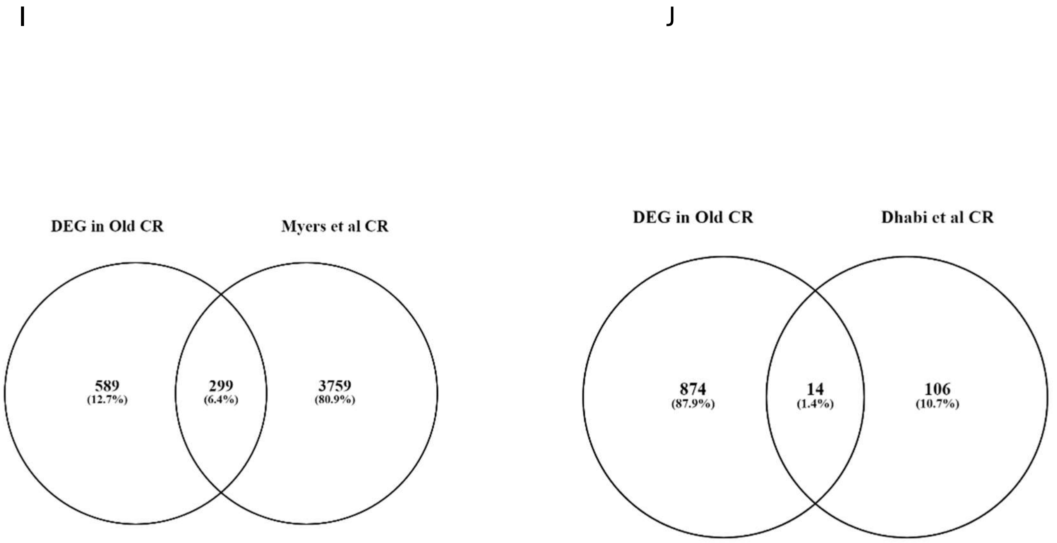

Figure S1. A. *Thirteen DEGs were present in both our study and a human study by Tumasian et al. (2021).* ***B.*** *Thirteen DEGs were present in both our study and a human study by Melov et al. (2007).* ***C.*** *Twenty-eight DEGs were present in both our study and a mouse muscle ageing study by Lin et al. (2018).* ***D.*** *Comparing our study with a single-cell RNA-seq study in Rattus norvegicus, 86 DEGs were present in both studies (Ma et al., 2020b).* ***E.*** *Comparing to a study on muscle ageing by Myers et al. (2021), we found 185 DEGs were present in both studies.* ***F.*** *Comparing our study with a meta-analysis of the ageing transcriptome (including muscle data) by Palmer et al. (2021), we found 60 DEGs present in both studies.* ***G.*** *Examining DEGs in relation to the Human Ageing Genomic Resources (HAGR) database (Tacutu et al., 2018), 21 DEGs were present in both our study and the database.* ***H.*** *Nine DEGs were present in both our DEGs found in old calorie-restricted rat muscle and previous human CR-associated DEGs (Das et al., 2023).* ***I.*** *Two hundred ninety-nine DEGs were present in both our study and another rat CR study on muscle ageing (Myers et al., 2021).* ***J.*** *Fourteen DEGs were present in both our study and a previous CR-treated study on muscle ageing (Dhahbi et al., 2012). Venn diagram was created using: https://bioinfogp.cnb.csic.es/tools/venny/.*

Figure S2. RNA quality analysis.

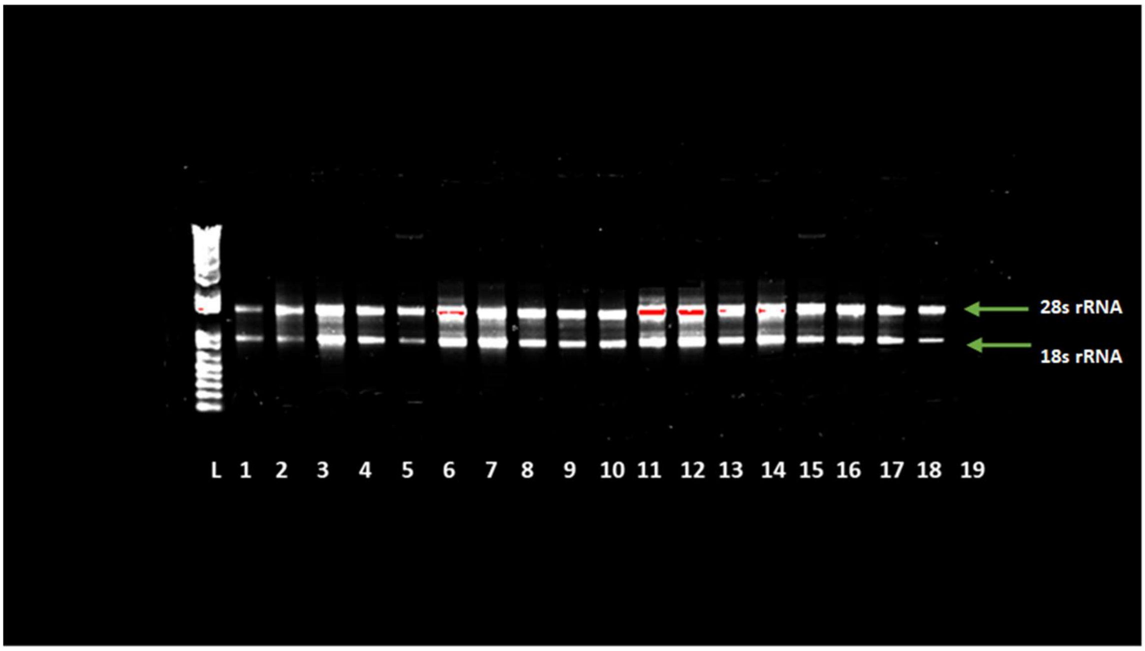

***Figure S2**. Quality analysis of RNA samples as observed in 1% agarose gel. All RNAs from all samples were intact and good quality. Lane L represents DNA ladder, Lanes 1-18 represents RNA samples and Lane 19 represents negative control.*
